# Association Between Preoperative Monocyte to High-Density Lipoprotein Ratio on In-hospital and Long-Term Mortality in Patients Undergoing Endovascular Repair for Acute Type B Aortic Dissection

**DOI:** 10.3389/fcvm.2021.775471

**Published:** 2022-01-07

**Authors:** Enmin Xie, Fan Yang, Songyuan Luo, Yuan Liu, Ling Xue, Wenhui Huang, Nianjin Xie, Lyufan Chen, Jitao Liu, Xinyue Yang, Sheng Su, Jie Li, Jianfang Luo

**Affiliations:** ^1^Department of Cardiology, Vascular Center, Guangdong Cardiovascular Institute, Guangdong Provincial Key Laboratory of Coronary Heart Disease Prevention, Guangdong Provincial People's Hospital, Guangdong Academy of Medical Sciences, Guangzhou, China; ^2^Graduate School of Peking Union Medical College, Chinese Academy of Medical Sciences, Beijing, China; ^3^Department of Emergency and Critical Care Medicine, Guangdong Provincial People's Hospital, Guangdong Academy of Medical Sciences, Guangzhou, China; ^4^The Second School of Clinical Medicine, Southern Medical University, Guangzhou, China

**Keywords:** biomarker, high-density lipoprotein, monocyte, prognosis, type B aortic dissection, thoracic endovascular aortic repair

## Abstract

**Aims:** The monocyte to high-density lipoprotein ratio (MHR), a novel marker of inflammation and cardiovascular events, has recently been found to facilitate the diagnosis of acute aortic dissection. This study aimed to assess the association of preoperative MHR with in-hospital and long-term mortality after thoracic endovascular aortic repair (TEVAR) for acute type B aortic dissection (TBAD).

**Methods:** We retrospectively evaluated 637 patients with acute TBAD who underwent TEVAR from a prospectively maintained database. Multivariable logistic and cox regression analyses were conducted to assess the relationship between preoperative MHR and in-hospital as well as long-term mortality. For clinical use, MHR was modeled as a continuous variable and a categorical variable with the optimal cutoff evaluated by receiver operator characteristic curve for long-term mortality. Propensity score matching was used to diminish baseline differences and subgroups analyses were conducted to assess the robustness of the results.

**Results:** Twenty-one (3.3%) patients died during hospitalization and 52 deaths (8.4%) were documented after a median follow-up of 48.1 months. The optimal cutoff value was 1.13 selected according to the receiver operator characteristic curve (sensitivity 78.8%; specificity 58.9%). Multivariate analyses showed that MHR was independently associated with either in-hospital death [odds ratio (OR) 2.11, 95% confidence interval (CI) 1.16-3.85, *P* = 0.015] or long-term mortality [hazard ratio (HR) 1.78, 95% CI 1.31-2.41, *P* < 0.001). As a categorical variable, MHR > 1.13 remained an independent predictor of in-hospital death (OR 4.53, 95% CI 1.44-14.30, *P* = 0.010) and long-term mortality (HR 4.16, 95% CI 2.13-8.10, *P* < 0.001). Propensity score analyses demonstrated similar results for both in-hospital death and long-term mortality. The association was further confirmed by subgroup analyses.

**Conclusions:** MHR might be useful for identifying patients at high risk of in-hospital and long-term mortality, which could be integrated into risk stratification strategies for acute TBAD patients undergoing TEVAR.

## Introduction

Acute type B aortic dissection (TBAD) is a life-threatening cardiovascular disease with high morbidity and mortality (1). Thoracic endovascular aortic repair (TEVAR) has become a valuable strategy for patients with TBAD if the anatomical conditions are appropriate (2). However, the short- and long-term postoperative mortality stay at a high level (3). Therefore, preoperative evaluation is necessary to identify patients at high risk to tailor the treatment options for each patient to improve prognosis.

Inflammation plays an important role in the pathological process of acute aortic dissection formation and is related to an increased risk for rupture and progression (1, 4). Monocytes are primary sources of the pro-inflammatory mediators (such as cytokines), and an elevation in monocytes might indicate a sub-clinical inflammation status (5, 6). In addition, high-density lipoprotein cholesterol (HDL-C) is a major component of total cholesterol (TC) with anti-inflammatory and protective functions (7). Therefore, an elevated monocyte to HDL-C ratio (MHR), reflecting either elevated monocytes or reduced HDL-C, or both, indicates homeostatic perturbations and sub-clinical inflammation (8). In recent years, elevated MHR has been revealed as a novel indicator for cardiovascular diseases and correlated with poor outcomes (8–11). For patients with acute aortic dissection, several studies have illustrated that medial recruitment and activation of monocytes/ macrophages and subsequent elastin degradation are initial events in the early stages (12–15), leading to a significant change in the numbers and phenotypes of monocytes (16). HDL-C was found to be inversely correlated with the monocyte counts in mice as well as children with familial hypercholesterolemia (17), and a lower HDL-C may increase the risk of aortic dissection (18). Recently, MHR was identified to have a diagnostic value in acute aortic dissection patients (19). However, far too little attention has been paid to the impact of preoperative MHR on prognosis in patients with acute TBAD undergoing TEVAR.

Thus, this study aimed to assess the association between preoperative MHR and in-hospital as well as long-term mortality after TEVAR for acute TBAD.

## Methods

### Patient Population

**Figure 1:**
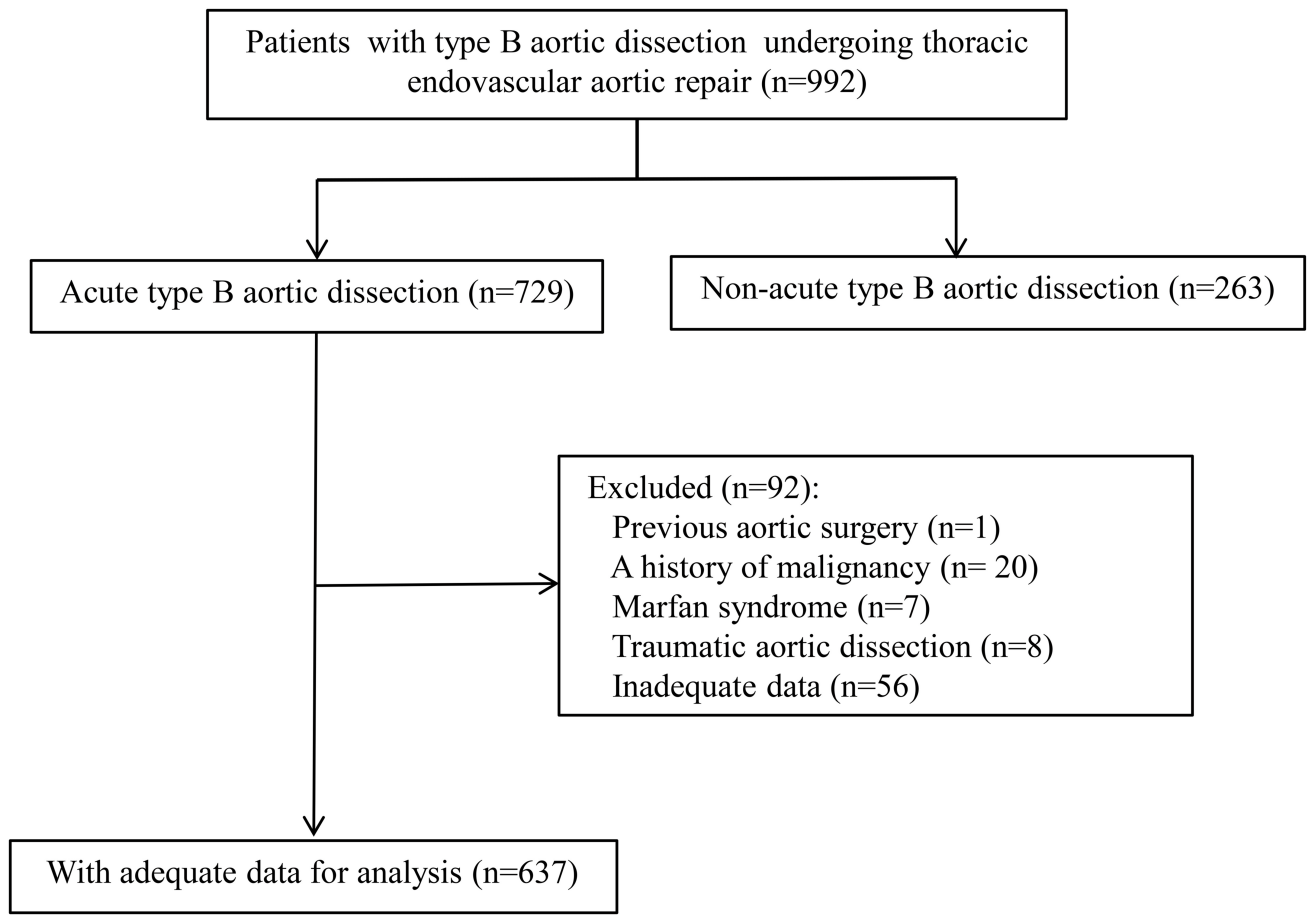
Flow diagram of the included population of acute type B aortic dissection patients treated with thoracic endovascular aortic repair.

A retrospective study of consecutive patients with acute TBAD undergoing TEVAR was conducted in Guangdong Provincial People's Hospital (Guangdong, China) between January 2010 to December 2017. Contrast-enhanced computed tomography angiography (CTA) was used to confirm the diagnosis of TBAD using Stanford classification criteria. Patients were excluded for the following criteria: (1) traumatic aortic dissection, (2) previous aortic surgery, (3) malignant tumor, (4) Marfan syndrome (confirmed preoperatively according to the Ghent criteria or revised Ghent criteria) (20, 21), (5) incomplete clinical data. The remaining 637 patients were included for a retrospective analysis (Figure 1). This research program was approved by the Ethics Committee of Guangdong Provincial People's Hospital with an informed consent waiver due to the retrospective study design.

### Procedure

Patients were treated with optimal medications and TEVAR as recommended by guidelines (2). The considerations for TEVAR in patients with uncomplicated TBAD included aortic diameter more than 40 mm, primary entry tear diameter more than 10 mm, a patent or partial thrombosed false lumen, and false lumen diameter more than 22 mm (22). The details of the standardized procedure for TEVAR had been described in our previous study (22). Briefly, the procedures were conducted in a cardiac catheterization room generally under local anesthesia. To cover the primary entry tear, stent-grafts were placed retrograde *via* femoral artery access using pre-closing method, and the diameter of the stent-graft was generally oversized by 5-10%. To obtain a 1.5-2 cm proximal landing zone, it was permitted to cover the origin of the left subclavian artery (LSA). The decision of reconstruction of the LSA was mainly determined by radiographic assessments of the vertebrobasilar circulation.

### Follow-Up and Data Collection

The information of survival and clinical manifestations were obtained through outpatient clinic interviews or telephone interviews. CTA was repeated at one, three, six, and 12 months following surgery, as well as annually thereafter. The follow-up imaging could be undertaken in any institution, and the results of reexaminations were assessed by two independent physicians. Baseline demographics, clinical characteristics, laboratory test results, imaging findings, medications, and outcomes were recorded on an electronic standardized form.

### Definitions

Acute TBAD was defined as a type B aortic dissection that occurred within 14 days of the onset of symptoms (2). Complicated TBAD was considered with the presence of any following: dissection with refractory pain, uncontrolled hypertension despite full medication, rapid aortic expansion, malperfusion syndromes, or signs of rupture (hemothorax, increasing periaortic, and mediastinal hematoma) (2).

Blood samples were collected from all patients following admission. Monocyte counts were measured by an automated hematological counter (XE-5000, Sysmex, Kobe, Japan). Levels of HDL-C were evaluated enzymatically from fasting venous blood, using an automated biochemical analyzing apparatus (UniCel DxC 800 Synchron, Beckman Coulter, Brea, CA, USA). MHR was derived by dividing monocyte counts (×10^9^/L) by HDL-C level (mmol/L). All laboratory tests were carried out in the study center's Laboratory Department using established measuring procedures (ISO 9000 Quality Management and Assurance Standards).

Outcomes were reported in accordance with the TBAD reporting standards (23). The primary outcomes of interest were in-hospital and long-term all-cause death. In-hospital death was defined as mortality occurring during the hospitalization after TEVAR. Long-term death was defined as mortality that occurred after discharge or more than 30 days after TEVAR. In-hospital major adverse clinical events (MACE), including death, stroke, spinal cord ischemia, limb or visceral ischemia, and re-intervention, were considered as the secondary outcomes of interest.

### Statistical Analysis

Continuous variables were presented as means ± standard deviation or median (quartiles 1 through 3) according to the distribution characteristics, and compared by student *t*-test or Mann-Whitney test. For categorical variables, data were expressed as percentages and performed by Chi-squared test or Fisher's exact test. Furthermore, we compared the level of MHR among different timing group based on time intervals from the symptom onset to admission according to the International Registry of Aortic dissection (IRAD) classification system for characterizing survival (24): ≤ 24 h, 2-7 days, and 8-14 days.

Cumulative survival curves were performed by Kaplan-Meier method, with log-rank tests used to discriminate between the curves of groups. The receiver operating characteristic curve (ROC) was constructed to evaluate the predictive validity of MHR for in-hospital and long-term all-cause mortality, and the area under curve (AUC) was compared by Delong's method. To assess the association between preoperative MHR and mortality, univariable and multivariable logistic regression models were built for in-hospital mortality, and cox regression models for long-term mortality. The candidate variables for the multivariable model were listed in Table 1. Variables with a *P*-value <0.1 in the univariable analysis were entered in the multivariable models using a forward stepwise approach. Odds ratio (OR) or hazard ratio (HR) and corresponding 95% confidence interval (CI) were reported. MHR was firstly entered as a continuous variable. Moreover, MHR was modeled as a categorical variable, with the optimal cutoff evaluated by ROC curve.

**Table 1:** Baseline characteristics stratified by preoperative monocyte to high-density lipoprotein ratio (MHR) before and after propensity score matching.

**Variables**	**Unmatched groups**				**Propensity score-matched groups**			
	**MHR ≤ 1.13 (*n* = 347)**	**MHR >1.13 (*n* = 290)**	**SMD**	***P-*value**	**MHR ≤ 1.13 (*n* = 190)**	**MHR > 1.13 (*n* = 190)**	**SMD**	***P-*value**
Age, years	54.5 ± 10.8	52.9 ± 11.3	0.138	0.083	53.0 ± 10.8	52.8 ± 11.1	0.022	0.833
Male sex	285 (82.1)	269 (92.8)	0.324	<0.001	178 (93.7)	170 (89.5)	0.152	0.139
BMI, kg/m^2^	24.7 ± 3.9	24.9 ± 3.6	0.039	0.625	24.4 ± 3.6	24.9 ± 3.7	0.117	0.257
Hypertension	299 (86.2)	248 (85.5)	0.019	0.815	165 (86.8)	166 (87.4)	0.016	0.878
Diabetes mellitus	20 (5.8)	14 (4.8)	0.042	0.601	9 (4.7)	9 (4.7)	<0.001	1.0
Hyperlipidemia	47 (13.5)	24 (8.3)	0.169	0.035	21 (11.1)	20 (10.5)	0.017	0.869
Coronary artery disease	46 (13.3)	44 (15.2)	0.055	0.489	31 (16.3)	28 (14.7)	0.043	0.671
Cerebrovascular disease	12 (3.5)	6 (2.1)	0.085	0.292	6 (3.2)	5 (2.6)	0.031	0.760
Smoke	173 (49.9)	155 (53.4)	0.072	0.366	111 (58.4)	101 (53.2)	0.106	0.302
Complicated TBAD	225 (64.8)	224 (77.2)	0.276	0.001	146 (76.8)	135 (71.1)	0.132	0.199
**Extent of dissection**			0.027	0.732			0.027	0.796
Confined in thoracic aorta	68 (19.6)	60 (20.7)			38 (20.0)	36 (18.9)		
Extended to abdominal aorta	279 (80.4)	230 (79.3)			152 (80.0)	154 (81.1)		
**False lumen patency**			0.196	0.033			0.068	0.400
Patent	249 (71.8)	186 (64.1)			130 (68.4)	139 (73.2)		
Partial thrombosed	90 (25.9)	88 (30.3)			54 (28.4)	43 (22.6)		
Completely thrombosed	8 (2.3)	16 (5.5)			6 (3.2)	8 (4.2)		
Maximum aortic diameter in lesion, mm	37.0 (33.4-41.6)	37.0 (34.0-43.0)	0.111	0.273	38.0 (35.0-42.0)	38.0 (34.0-44.1)	0.074	0.616
Pleural effusion	150 (43.2)	154 (53.1)	0.198	0.013	88 (46.3)	90 (47.4)	0.021	0.837
Pericardial effusion	11 (3.2)	16 (5.5)	0.115	0.143	7 (3.7)	7 (3.7)	<0.001	1.0
WBC count, 10^9^/L	10.7 (8.7-12.7)	12.2 (10.0-14.8)	0.453	<0.001	11.0 (9.1-13.6)	11.6 (9.7-13.9)	0.079	0.183
Monocyte count, 10^9^/L	0.8 (0.6-1.0)	1.3 (1.1-1.6)	1.558	<0.001	0.7 (0.6-1.0)	1.3 (1.1-1.5)	1.651	<0.001
Hemoglobin, g/L	130.3 (118.0-141.0)	133.0 (120.0-141.9)	0.031	0.378	131.0 (119.0-140.0)	133.0 (118.1-142.2)	0.026	0.529
Platelet, 10^9^/L	192.0 (155.0-242.0)	208.0 (161.6-291.0)	0.287	0.002	196.7 (155.8-258.3)	199.8 (160.7-263.5)	0.058	0.568
Serum creatinine, mg/dl	1.0 (0.8-1.3)	1.1 (0.9-1.5)	0.092	0.013	1.1 (0.9-1.6)	1.1 (0.9-1.4)	0.085	0.130
D-Dimer, μg/ml	2.5 (1.1-4.2)	2.3 (0.8-4.0)	0.096	0.169	2.3 (0.9-4.0)	2.4 (0.9-4.2)	0.011	0.866
TC, mmol/L	4.6 (3.9-5.2)	4.2 (3.5-4.8)	0.374	<0.001	4.3 (3.6-5.0)	4.5 (3.8-5.0)	0.007	0.612
TG, mmol/L	1.3 (1.0-1.8)	1.3 (0.9-1.7)	0.067	0.367	1.3 (1.0-1.8)	1.3 (0.9-1.7)	0.005	0.761
LDL-C, mmol/L	2.7 (2.2-3.3)	2.5 (2.1-3.1)	0.219	0.014	2.6 (2.1-3.2)	2.6 (2.2-3.2)	0.027	0.517
HDL-C, mmol/L	1.1 (0.9-1.3)	0.8 (0.7-1.0)	0.870	<0.001	1.0 (0.9-1.2)	0.9 (0.7-1.0)	0.658	<0.001
ALT, U/L	20.0 (14.0-32.0)	25.0 (16.5-45.2)	0.227	<0.001	20.7 (15.0-34.7)	24.0 (15.8-41.0)	0.027	0.104
AST, U/L	20.5 (16.6-27.6)	24.4 (19.0-39.0)	0.221	<0.001	21.0 (17.0-32.0)	24.0 (18.5-34.0)	0.057	0.092
**Medications at admission**
Antiplatelet drugs	63 (18.2)	36 (12.4)	0.160	0.046	31 (16.3)	30 (15.8)	0.014	0.889
ACEI	78 (22.5)	58 (20.0)	0.061	0.447	41 (21.6)	35 (18.4)	0.079	0.442
ARB	165 (47.6)	127 (43.8)	0.075	0.343	85 (44.7)	87 (45.8)	0.021	0.837
Beta-blockers	326 (93.9)	270 (93.1)	0.034	0.665	176 (92.6)	178 (93.7)	0.042	0.684
Calcium channel blockers	273 (78.7)	233 (80.3)	0.041	0.603	149 (78.4)	150 (78.9)	0.013	0.900
Statins	150 (43.2)	131 (45.2)	0.039	0.623	84 (44.2)	81 (42.6)	0.032	0.756

*Values are given as number (percentage) or mean ± SD. ACEI, angiotensin-converting enzyme inhibitors; ALT, alanine aminotransferase; ARB, angiotensin receptor blockers; AST, aspartate aminotransferase; BMI, body mass index; HDL-C, high density lipoprotein cholesterol; LDL-C, low density lipoprotein cholesterol; MHR, monocyte to high-density lipoprotein ratio; SMD, standardized mean difference; TBAD, type B aortic dissection; TC, total cholesterol; TG, triglycerides; WBC, white blood cell*.

To adjust for baseline variations and diminish selection bias, a propensity score matching (PSM) analysis was conducted using a 1:1 nearest-neighbor matching with a caliper of 0.01. Propensity score was calculated for each patient based on a logistic regression model using variables listed in Table 1. The difference between groups after PSM were measured by standardized mean difference (SMD). A maximum SMD of 0.1, or even 0.15, is usually regarded as appropriate. Moreover, we categorized patients into three groups by the tertile of MHR to enhance clinical utility, and repeated the analyses. To investigate the consistency of the conclusion, subgroup analyses were performed by age (<65 vs. **≥**65 years), gender (male vs. female), severity of disease (complicated vs. uncomplicated), anemia status (yes vs. no), and kidney function (eGFR <60 vs. eGFR ≥ 60 ml/min/1.73 m^2^). Subgroup analyses were not performed for in-hospital death because of the low mortality.

A two-tailed *P*-value <0.05 was considered statistically significant. All statistical analyses were carried out using the SPSS 23.0 (IBM SPSS 23 Inc) and R software (version 3.5.1).

## Results

### Baseline Characteristics

Among 637 enrolled patients, the mean age was 54.0 ± 11.1 years, and 554 (87.0%) were male. The average BMI 24.8 ± 3.8 kg/m^2^, and the most common comorbidity was hypertension (85.9%). The median (interquartile range) of preoperative MHR was 1.09 (0.77-1.44), ranging from 0.02 to 4.78. Compared with uncomplicated cases, MHR was significantly higher in patients with acute complicated TBAD (1.13 [0.82-1.46] vs. 0.93 [0.70-1.39], *p* = 0.007). Moreover, patients with MHR > 1.13 were more likely to have a greater percentage of complicated patients compared with those with MHR ≤ 1.13 (77.2 vs. 64.8%, *P* = 0.001, Table 1). Regarding different timing groups, MHR was significantly higher in the 8-14 days group compared with 2-7 days and ≤ 24 h group (1.19 [0.80-1.66] vs. 1.01 [0.83-1.45] vs. 0.96 [0.65-1.36], *P* = 0.001]). Subgroup analyses showed that MHR was still significantly higher in the 8-14 days group compared with other two timing groups in complicated cases (1.24 [0.85-1.76] vs. 1.15 [0.88-1.49] vs. 0.97 [0.68-1.38], *P* = 0.001), while the differences among three timing groups in uncomplicated cases were not statistically significant (1.13 [0.74-1.51] vs. 0.90 [0.73-1.36] vs. 0.92 [0.55-1.19], *P* = 0.180). Demographic and clinical characteristics were shown in Table 1. Patients were classified into two groups using a cutoff of 1.13 (sensitivity 78.8%; specificity 58.9%) calculated based on the ROC curve for long-term mortality. In the entire cohort, patients with MHR > 1.13 were more likely to be male, with a higher percentage of complicated patients or pleural effusion, and with a lower percentage of hyperlipidemia history or patent false lumen. In addition, patients with MHR > 1.13 had higher preoperative WBC counts, platelet counts, serum creatinine, alanine aminotransferase (ALT) as well as aspartate aminotransferase (AST), and lower levels of TC as well as low density lipoprotein cholesterol (LDL-C).

### In-hospital Outcomes

**Figure 2:**
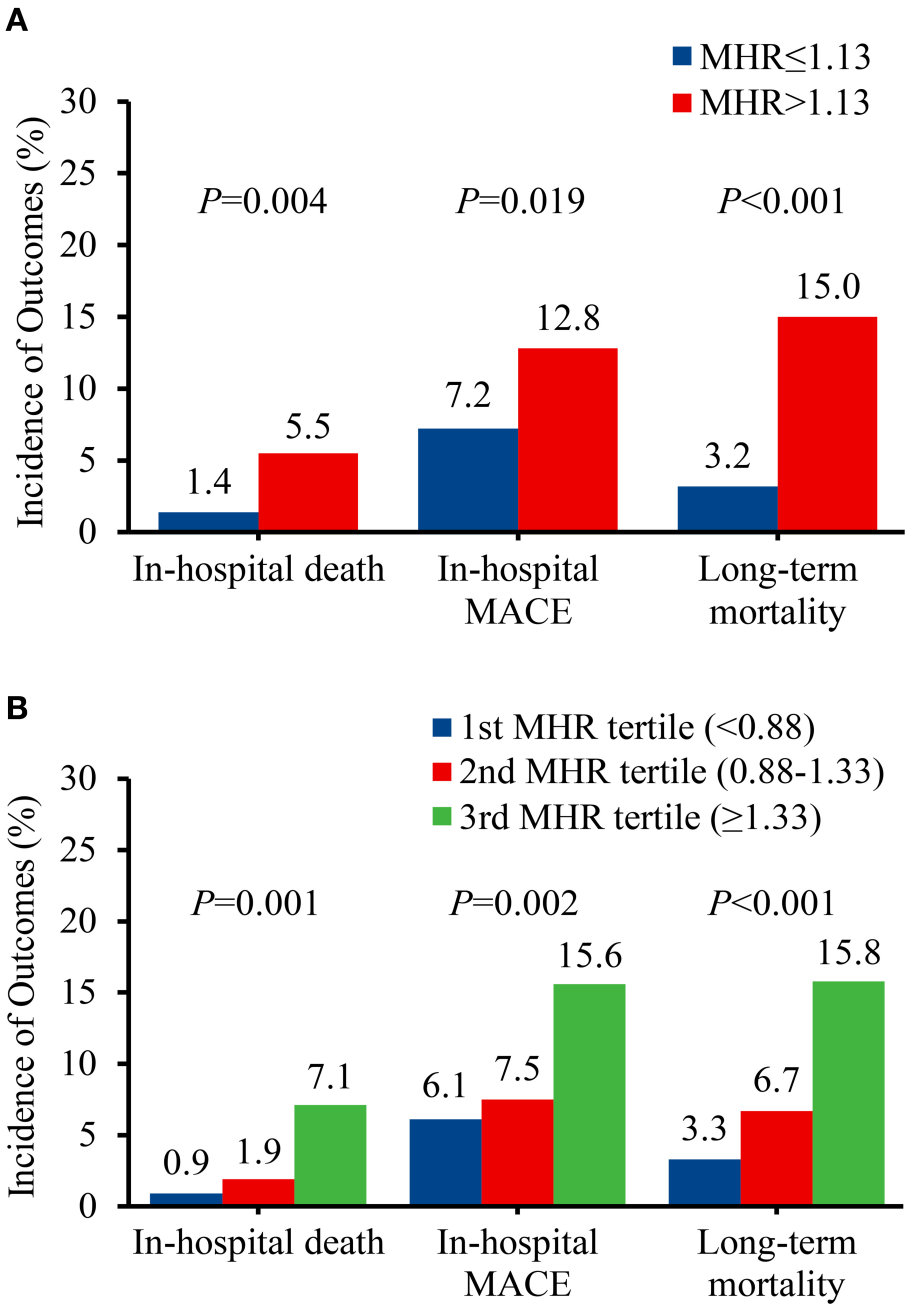
Prevalence of adverse events before propensity score matching. **(A)** Classified by the receiver operating characteristic curve selected cut-off of MHR. **(B)** Classified by the tertiles of MHR. MACE, major adverse clinical events; MHR, monocyte to high-density lipoprotein ratio.

In total, 21 (3.3%) in-hospital deaths were recorded, with 16 patients presented with MHR > 1.13. The incidence of in-hospital death was significantly higher in patients with MHR > 1.13 than that with MHR ≤ 1.13 (5.5 vs. 1.4%, *P* = 0.004) (Figure 2A). During the hospitalization period, 21 (3.3%) patients had stroke, 7 (1.1%) patients had spinal cord ischemia, 14 (2.2%) patients had limb ischemia, 3 (0.5%) patients had visceral ischemia, and 9 (1.4%) patients had re-intervention. The rates of in-hospital MACE were significantly higher in patients with MHR > 1.13 than that with MHR ≤ 1.13 (12.8 vs. 7.2%, *P* = 0.019) (Figure 2A). Multivariable logistic analysis revealed that MHR (modeled as a continuous variable) was associated with a significantly increased risk of in-hospital mortality [odds ratio (OR) 2.11, 95% confidence interval (CI) 1.16-3.85, *P* = 0.015) (Table 2). Other independent predictors for in-hospital death were maximum aortic diameter, hemoglobin, AST, and calcium channel blockers (Supplementary Table 1). Though the cut-off valve of 1.3 was generated from follow-up deaths, we found a significant association between MHR > 1.13 and in-hospital mortality (OR 4.53, 95% CI 1.44-14.30, *P* = 0.010) (Table 2 and Supplementary Table 1).

**Table 2:** Association of preoperative monocyte to high-density lipoprotein ratio (MHR) on in-hospital and long-term death before and after propensity score matching.

**Variables**	**Unmatched groups**				**Propensity score-matched groups**			
	**Continuous MHR**	***P***	**>1.13 vs. ≤1.13**	***P***	**Continuous MHR**	***P***	**>1.13 vs. ≤1.13**	***P***
**In-hospital death**
Unadjusted OR (95%CI)	2.00 (1.22*-*3.30)	0.006	3.99 (1.45-11.04)	0.008	2.18 (1.14-4.18)	0.018	4.20 (1.17-15.14)	0.028
Adjusted OR (95% CI)^*^	2.11 (1.16-3.85)	0.015	4.53 (1.44-14.30)	0.010	2.72 (1.23-5.99)	0.013	7.12 (1.52-33.45)	0.013
**Long-term death**				
Unadjusted HR (95%CI)	1.68 (1.26-2.25)	<0.001	4.27 (2.19-8.32)	<0.001	1.77 (1.21-2.58)	0.003	3.89 (1.78-8.53)	0.001
Adjusted HR (95% CI)^*^	1.78 (1.31-2.41)	<0.001	4.16 (2.13-8.10)	<0.001	1.87 (1.27-2.76)	0.002	4.02 (1.83-8.83)	0.001

* *Covariates for the multivariable model include age, gender, body mass index, hypertension, diabetes mellitus, hyperlipidemia, coronary artery disease, cerebrovascular disease, smoke, complicated (vs. uncomplicated), extended to abdominal aorta (vs. confined in thoracic aorta), false lumen status, maximum aortic diameter in lesion, pleural effusion, pericardial effusion, white blood cell count, monocyte count, hemoglobin, platelet, serum creatinine, D-Dimer, total cholesterol, triglycerides, low density lipoprotein cholesterol, high density lipoprotein cholesterol, alanine aminotransferase, aspartate aminotransferase, antiplatelet drugs, angiotensin-converting enzyme inhibitors, angiotensin receptor blockers, beta-blockers, calcium channel blockers, and statins. Variables with a p-value <0.1 in univariable analysis were entered in the multivariable models. CI, confidence interval; HR, hazard ratio; MHR, monocyte to high-density lipoprotein ratio; OR, odds ratio*.

### Survival Analysis

**Figure 3:**
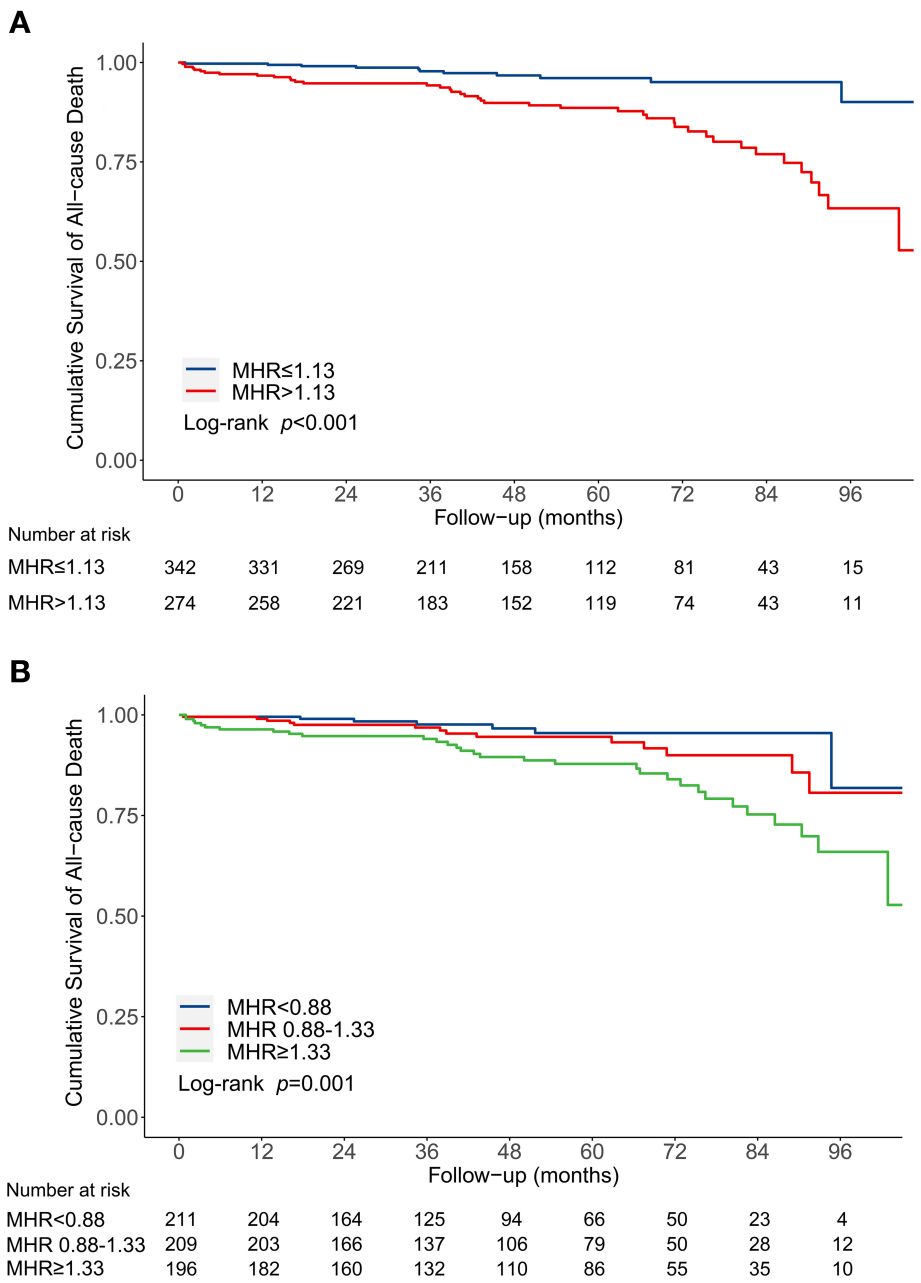
Kaplan-Meier curve for cumulative survival rates of long-term mortality before propensity score matching. **(A)** Classified by the receiver operating characteristic curve selected cut-off of MHR. **(B)** Classified by the tertiles of MHR. MHR, monocyte to high-density lipoprotein ratio.

Following a median follow-up of 48.1 months (interquartile range, 26.2-72.2 months), 52 deaths (8.4%) were documented after discharge, with 41 patients in MHR > 1.13 group. The incidence of long-term mortality was significantly higher in patients with MHR > 1.13 than that with MHR ≤ 1.13 (15.0 vs. 3.2%, *P* < 0.001) (Figure 2A). Multivariable Cox regression analysis indicated that MHR (modeled as a continuous variable) was independently associated with long-term mortality [hazard ratio (HR) 1.78, 95% CI 1.31-2.41, *P* < 0.001] (Table 2). Other independent predictors for long-term mortality included maximum aortic diameter, coronary artery disease history, and serum creatinine (Supplementary Table 2). As a categorical variable, MHR > 1.13 was associated with a significantly increased risk of long-term mortality (HR 4.16, 95% CI 2.13-8.10, *P* < 0.001) (Table 2 and Supplementary Table 2). Patients with MHR > 1.13 had a significantly greater cumulative long-term mortality risk than patients with MHR ≤ 1.13. (log-rank = 21.66, *P* < 0.001) (Figure 3A).

### Propensity Score Matched Analysis

After PSM, 190 matched pairs were obtained. The baseline characteristics in the matched cohort were outlined in Table 1. In-hospital mortality rate remained significantly higher for patients with MHR > 1.13 in the matched cohort (6.3 vs. 1.6%, *P* = 0.018), while the rates of in-hospital MACE were comparable between two matched groups (13.2 vs. 9.5%, *P* = 0.257) (Supplementary Figure 1). Either as a continuous variable (OR 2.72, 95% CI 1.23-5.99, *P* = 0.013) or as a categorical variable (OR 7.12, 95% CI 1.52-33.45, *P* = 0.013), the association between MHR and in-hospital death persisted in the multivariable logistic analysis after matching (Table 2 and Supplementary Table 3). In survival analyses, long-term mortality rate remained significantly higher for patients with MHR >1.13 in the matched cohort (16.3 vs. 4.3%, *P* < 0.001) (Supplementary Figure 1). Multivariable Cox regression analyses showed that MHR, modeled as either a continuous variable (HR 1.87, 95% CI 1.27-2.76, *P* = 0.002) or a categorical variable (more than 1.13) (HR 4.02, 95% CI 1.83-8.83, *P* = 0.001), was an independent predictor for long-term mortality after matching (Table 2 and Supplementary Table 4). Patients with MHR > 1.13 had a significantly greater cumulative long-term mortality risk than patients with MHR ≤ 1.13 (log-rank = 13.37, *P* < 0.001) (Supplementary Figure 2).

### Diagnostic Accuracy and Clinical Utility of MHR

**Figure 4:**
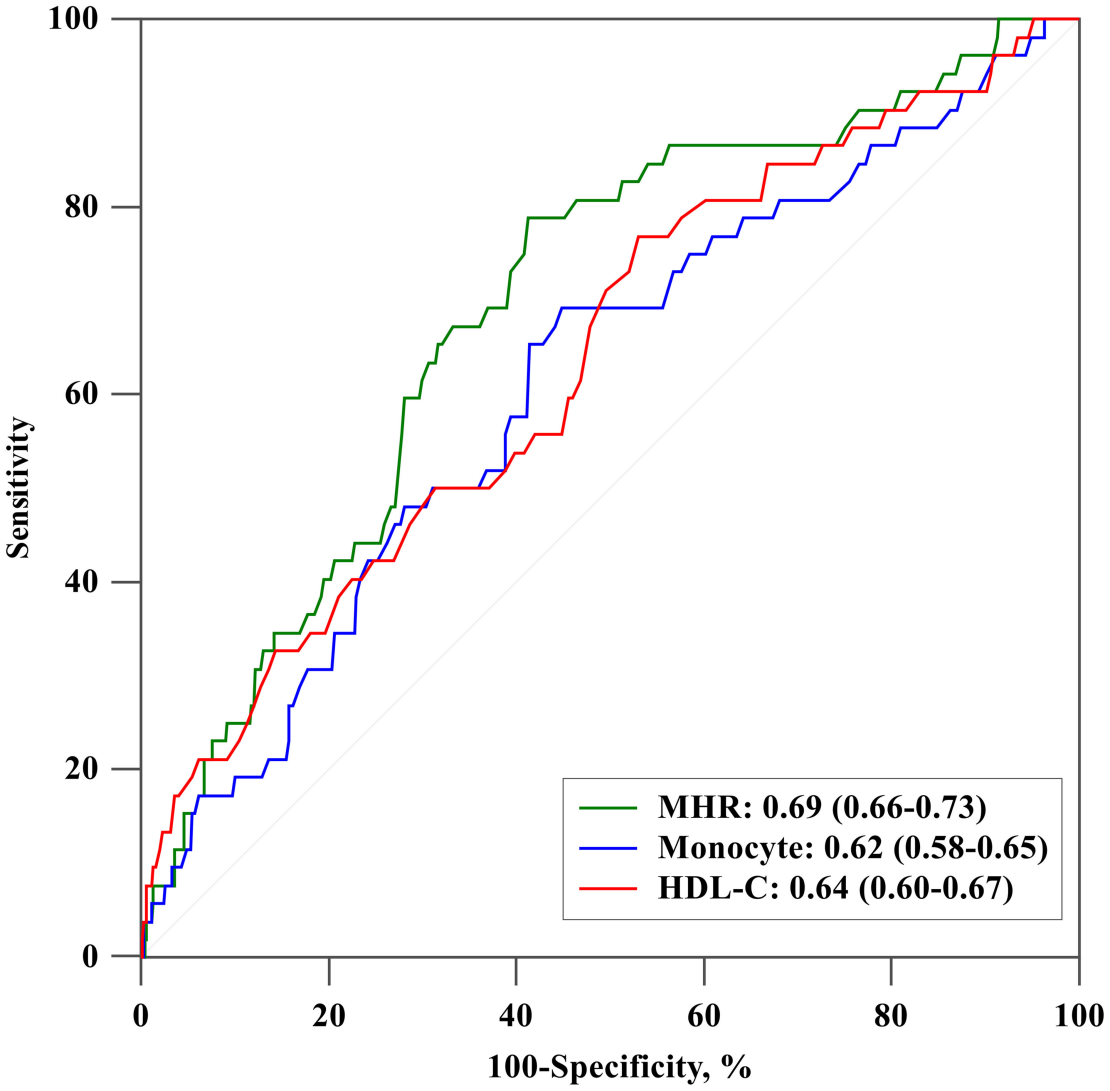
ROC curves of MHR, monocyte, and HDL-C for long-term mortality. HDL-C, high-density lipoprotein cholesterol. MHR, monocyte to high-density lipoprotein ratio.

The predictive value of monocyte count, HDL-C, and MHR for long-term mortality was determined using a ROC curve analysis. Figure 4 showed that MHR (AUC 0.694, 95% CI 0.656-0.730, *P* < 0.001) was superior to either monocyte count (AUC 0.615, 95% CI 0.575-0.654, *P* = 0.006) or HDL-C (AUC 0.635, 95% CI 0.596-0.674, *P* = 0.001) (compared with monocyte count and HDL-C: *P* = 0.008 and 0.176, respectively).

We categorized patients into three groups by the tertile of MHR to enhance clinical utility: <0.88, 0.88-1.33, and ≥1.33. The in-hospital mortality (0.9 vs. 1.9 vs. 7.1%, *P* = 0.001), in-hospital MACE (6.1 vs. 7.5 vs. 15.6%, *P* = 0.002), and long-term mortality (3.3 vs. 6.7 vs. 15.8%, *P* < 0.001) increased from the first to third MHR tertile (Figure 2B). Compared with the lowest MHR tertile, the highest MHR tertile was independently associated with in-hospital mortality (adjusted OR 12.43, 95% CI 2.17-71.11, *P* = 0.005) (Supplementary Table 1) and long-term mortality (adjusted HR 3.78, 95% CI 1.66-8.60, *P* = 0.002) (Supplementary Table 2). The cumulative long-term mortality risks among the three groups were significantly different (log-rank = 15.02, *P* = 0.001) (Figure 3B).

In subgroup analyses, we stratified the patients by age (<65 vs. **≥**65 years), gender (male vs. female), severity of disease (complicated vs. uncomplicated), anemia status (yes vs. no), and kidney function (eGFR <60 vs. eGFR ≥ 60 ml/min/1.73 m^2^). Multivariable Cox regression analyses revealed that MHR, modeled as either a continuous or categorical (more than 1.13) variable, remained independently associated with long-term mortality in subgroups analyses (Table 3 and Supplementary Tables 5-14).

**Table 3:** Association of preoperative monocyte to high-density lipoprotein ratio (MHR) on long-term death in subgroups after multivariable adjustment^*^.

**Variables**	**Continuous MHR**		**>1.13 vs**. **≤1.13**	
	**HR (95%CI)**	***P***	**HR (95%CI)**	***P***
**Age groups, yrs**			
<65	1.61 (1.13-2.28)	0.008	3.92 (1.82-8.42)	<0.001
≥65	3.49 (1.66-7.35)	0.001	8.16 (1.78-37.42)	0.007
**Gender group**			
Male	1.69 (1.23-2.33)	0.001	3.53 (1.77-7.03)	<0.001
Female	17.16 (1.69-174.82)	0.016	235.84 (0.04-1.51E + 6)	0.222
**Severity of disease**			
Uncomplicated	3.06 (1.31-7.20)	0.010	4.79 (1.26-18.17)	0.021
Complicated	1.34 (0.87-2.06)	0.185	4.64 (2.12-10.18)	<0.001
**Anemia**			
No	2.25 (1.33-3.82)	0.003	5.83 (1.70-20.06)	0.005
Yes	1.43 (0.86-2.37)	0.165	3.66 (1.60-8.38)	0.002
**eGFR** **<** **60 ml/min/1.73 m**^**2**^			
No	1.76 (1.08-2.88)	0.025	3.59 (1.53-8.47)	0.003
Yes	1.79 (1.22-2.61)	0.003	8.91 (2.82-28.16)	<0.001

* *Details in the Supplementary Tables 5–14. Covariates for the multivariable model include age, gender, body mass index, hypertension, diabetes mellitus, hyperlipidemia, coronary artery disease, cerebrovascular disease, smoke, complicated (vs. uncomplicated), extended to abdominal aorta (vs. confined in thoracic aorta), false lumen status, maximum aortic diameter in lesion, pleural effusion, pericardial effusion, white blood cell count, monocyte count, hemoglobin, platelet, serum creatinine, D-Dimer, total cholesterol, triglycerides, low density lipoprotein cholesterol, high density lipoprotein cholesterol, alanine aminotransferase, aspartate aminotransferase, antiplatelet drugs, angiotensin-converting enzyme inhibitors, angiotensin receptor blockers, beta-blockers, calcium channel blockers, and statins. Variables with a p-value <0.1 in univariable analysis were entered in the multivariable models. CI, confidence interval; eGFR, estimated glomerular filtration rate; HR, hazard ratio; MHR, monocyte to high-density lipoprotein ratio*.

## Discussion

The present study demonstrated that patients with elevated MHR experienced higher rates of in-hospital and long-term mortality among acute TBAD patients undergoing TEVAR. Multivariable analyses revealed that preoperative MHR was independently associated with in-hospital and long-term all-cause mortality. After PSM, the independent association between MHR and in-hospital as well as long-term mortality persisted. The association was further confirmed by subgroup analyses.

Inflammation, which involves monocytes and macrophages, was critical in the pathophysiology of aortic dissection (1, 4). Recruitment and activation of multiple inflammatory cells could lead to apoptosis of vascular smooth muscle cells, thus inducing wall fragility and subsequent aortic dilation, dissection, and even rupture (1, 25). In this condition, the numbers and phenotypes of monocytes were significantly altered (16, 26). A previous study demonstrated that classical monocytes were significantly increased in patients among the acute aortic dissection group compared with patients with carotid artery stenosis or subjects with traditional cardiovascular risk factors (26). Du et al. also reported similar findings, compared with healthy controls, in a Chinese Han population (19). Another study on acute TBAD patients found that the percentage of CD14^+^ monocytes was significantly higher than that of normal volunteers, in which the percentages of the two monocyte subsets (CD14^bright^CD16^−^ and CD14^bright^CD16^+^) were increased significantly (*P* < 0.001) (16). Furthermore, selective depletion of monocyte/macrophage in mice model dramatically reduced the incidence of aortic dissection, supporting that monocytes/ macrophages have an important role in the pathophysiology of aortic dissection (27).

Dyslipidemia was commonly observed in patients with aortic dissection. Previous literature found that patients with acute aortic dissection presented with a significantly lower level of total cholesterol as well as HDL-C, compared with healthy controls (19). By recruiting 30,412 individuals without aortic disease at baseline from a contemporary population, Landenhed and colleagues revealed that low apoA1 was significantly associated with incident aortic dissection during a 20-year follow-up period (28). The mechanisms of dyslipidemia among aortic dissection patients are still uncertain. One plausible explanation is that the impact of inflammation on the ability of HDL to mediate reverse cholesterol transport may promote the progress of fatty plaques and subsequent aortic dissection, as has been observed in many inflammation-related diseases (29, 30). In addition, the role of HDL-C as an anti-inflammatory agent in the prevention of cardiovascular disease has been documented, which may be partly explained by suppressing the activation of monocytes and proliferation–differentiation of monocyte progenitor cells (31, 32).

The integrated maker, MHR, has been considered a potential biomarker for cardiovascular diseases and correlated with poor outcomes, such as acute coronary syndrome (9, 10), atrial fibrillation recurrence (11), and abdominal aortic aneurysm (33). Moreover, Yayla et al. (34) indicated that MHR was able to independently predict aortic distensibility and aortic stiffness among newly diagnosed untreated patients with hypertension. A previous study including 128 acute aortic dissection patients and 110 healthy controls demonstrated that baseline MHR levels were significantly higher in patients with acute aortic dissection, and a cutoff value of MHR > 0.37 was associated with acute aortic dissection with a sensitivity of 86.70% and a specificity of 93.60% (19). The present study further assessed the prognosis value of MHR on mortality in patients with acute TBAD undergoing TEVAR. Our results demonstrated that preoperative MHR, as a continuous predictor or as a categorical predictor (cutoff 1.13), was independently associated with in-hospital and long-term all-cause mortality before and after PSM. Replication of the results in subgroup analyses adds to the confidence that the results are reliable. In addition, our results support that MHR, a ratio combining the pro-inflammatory effects of monocytes and the anti-inflammatory effects of HDL, was a more powerful predictor than either parameter alone. Moreover, individuals with increased MHR had higher serum creatinine and hepatic enzyme levels, confirming earlier results that inflammation, in combination with aortic dissection, might affect organ function and exacerbate malperfusion (1). These results are in line with the findings in literature that elevated MHR may help identify patients at higher risk of poor prognosis in other cardiovascular settings (8–11, 33). Considering the heterogeneity in clinical characteristics and prognosis between acute and sub-acute patients, the present study only included acute TBAD patients. Moreover, the small sample size of non-acute TBAD patients during the study period in our center may make the analyses subject to potential error. Further studies are warranted to identify the association between MHR and non-acute TBAD patients treated with TEVAR.

Mounting evidence indicates that changes in inflammatory biomarkers, such as white blood cell (WBC) count, C-reactive protein (CRP), and D-dimer, are associated with acute-phase reactions as well as adverse events in acute aortic dissection patients (35–37). Nevertheless, most of these studies are based on limited numbers of patients and/or short follow-up periods, and a single inflammatory biomarker may be more affected by other factors such as drugs and immune status. The present study found that WBC, monocyte, and D-dimer all had an unadjusted increased risk of in-hospital mortality (OR 1.12 [*P* = 0.011], OR 2.29 [*P* = 0.052], and OR 1.08 [*P* = 0.016], respectively) (Supplementary Table 1), which was consistent with previous findings (36, 37). Multivariable logistic model showed that MHR, not WBC, monocyte, or D-dimer, was an independent predictor of in-hospital mortality, indicating that the prognostic value of MHR on in-hospital mortality may be superior to these inflammatory biomarkers (modeled as a continuous predictor: OR 2.11, 95% CI 1.16-3.85, *P* = 0.015; modeled as a categorical predictor with a cutoff 1.13: OR 4.53, 95% CI 1.44-14.30, *P*=0.010, respectively). After a median 48.1 months follow-up, similar results were observed among the analyses of the association between MHR and long-term mortality (modeled as a continuous predictor: HR 1.78, 95% CI 1.31-2.41, *P* < 0.001; as a categorical predictor with a cutoff 1.13: HR 4.16, 95% CI 2.13-8.10, *P* < 0.001, respectively). Taken together, MHR, a particular fraction with integration of pro-inflammatory and anti-inflammatory indices, might be useful for identifying patients at high risk of in-hospital and long-term mortality when performing TEVAR for acute TBAD.

Furthermore, subgroup analyses showed that MHR was independently associated with an increased risk of long-term mortality among uncomplicated patients. Currently, the aim of TEVAR for acute uncomplicated TBAD is to prevent late aortic related complications (2). The primary commitment is to ensure patient safety. These results should undoubtedly raise more attention in TEVAR for acute uncomplicated TBAD patients. Further research focusing on the impact of MHR on outcomes among these patients is warranted in the future.

The present study highlights the value of preoperative MHR on identifying patients who are at high risk of in-hospital and long-term mortality, suggesting that MHR might be suitable to be a risk assessment tool in clinical practice. High MHR might request more strict preoperative preparation and closer monitor and therefore to avoid adverse outcomes. Of note, the area under the AUC curve was 0.694, indicating that MHR alone may be insufficient for diagnosis and that other indicators should be included. As an obtainable and cost-effective biomarker to predict poor outcomes, MHR should be incorporated into risk stratification strategies when tailoring monitoring protocols.

## Limitations

There were several important limitations in this study. First, our study had selection bias for the native feature of a single-center retrospective design. Moreover, the mechanism between MHR and poor prognosis was not identified as it was an observational study. Second, MHR was not dynamically monitored so that we cannot determine whether MHR increased over time or whether changes in MHR were associated with the prognosis of TBAD patients. Third, since TEVAR was administered to all participants in this research, the effect of MHR on acute TBAD patients receiving best medical therapy, particularly those with uncomplicated cases, was not definite. Moreover, the patients included in this study were from a cohort representative of the TBAD in the Chinese population. More details and extensive prospective investigations are needed in the future to generalize the role of MHR in TBAD patients.

## Conclusion

This study demonstrated that preoperative MHR was independently associated with short- and long-term mortality in patients with acute TBAD undergoing TEVAR, suggesting the role of MHR in the risk stratification strategies. More attention should be raised on acute TBAD patients with preoperative high MHR.

## Data Availability Statement

The original contributions presented in the study are included in the article/Supplementary Material, further inquiries can be directed to the corresponding author/s.

## Ethics Statement

The studies involving human participants were reviewed and approved by Ethics Committee of Guangdong Provincial People's Hospital. Written informed consent for participation was not required for this study in accordance with the national legislation and the institutional requirements.

## Author Contributions

JLuo, JLi, and EX: research idea and study design. FY, SL, YL, LX, WH, NX, and LC: data collection and analysis. EX, JLiu, XY, and SS: statistical analysis. EX, FY, and SL: manuscript preparation. All authors: critical revision of manuscript.

## Funding

This work was supported by the High-Level Hospital Construction Project (grant number DFJH201807), Guangdong Provincial Clinical Research Center for Cardiovascular Disease (grant number 2020B1111170011), and Guangdong Provincial People's Hospital Clinical Research Fund (grant number Y012018085). The founders were not involved in the design of the study, data collection, data analysis, or preparation of the manuscript.

## Conflict of Interest

The authors declare that the research was conducted in the absence of any commercial or financial relationships that could be construed as a potential conflict of interest.

## Publisher's Note

All claims expressed in this article are solely those of the authors and do not necessarily represent those of their affiliated organizations, or those of the publisher, the editors and the reviewers. Any product that may be evaluated in this article, or claim that may be made by its manufacturer, is not guaranteed or endorsed by the publisher.

## Supplementary Material

Supplementary Figure 1 Prevalence of adverse events after propensity score matching. MACE, major adverse clinical events; MHR, monocyte to high-density lipoprotein ratio.

Supplementary Figure 2 Kaplan-Meier curve for cumulative survival rates of long-term mortality after propensity score matching. MHR, monocyte to high-density lipoprotein ratio.
