# Supplementary material for: Association Between Preoperative Monocyte to High-Density Lipoprotein Ratio on In-hospital and Long-Term Mortality in Patients Undergoing Endovascular Repair for Acute Type B Aortic Dissection
Source: Front Cardiovasc Med. 2022 Jan 7;8:775471. doi: 10.3389/fcvm.2021.775471 (PMC8777016; doi:10.3389/fcvm.2021.775471)
Supplement: Supplementary file 1 [file Data_Sheet_1.docx]

**Supplementary Table 1.** **Details of logistic regression analysis for in-hospital mortality before propensity score matching**

| **Variables** | **Univariate** | |  | **Multivariate** | | | | | | | |  |  |
| --- | --- | --- | --- | --- | --- | --- | --- | --- | --- | --- | --- | --- | --- |
|  | **OR** | ***P*** |  | **Continuous MHR** | |  | **>1.13 vs** ≤**1.13** | |  | **Tertile** | |  |  |
|  |  |  |  | **OR (95% CI)** | ***P*** |  | **OR (95% CI)** | ***P*** |  | **OR (95% CI)** | ***P*** |  |  |
| Continuous MHR | 2.00 | 0.006 |  | 2.11 (1.16-3.85) | 0.015 |  |  |  |  |  |  |  |  |
| MHR, >1.13 vs ≤1.13 | 3.99 | 0.008 |  |  |  |  | 4.53 (1.44-14.30) | 0.010 |  |  |  |  |  |
| MHR |  |  |  |  |  |  |  |  |  |  |  |  |  |
| Tertile 1, <0.88 | Ref | Ref |  |  |  |  |  |  |  | Ref | Ref |  |  |
| Tertile 2, 0.88-1.33 | 2.02 | 0.420 |  |  |  |  |  |  |  | 3.47 (0.52-23.37) | 0.201 |  |  |
| Tertile 3, ≥1.33 | 8.07 | 0.006 |  |  |  |  |  |  |  | 12.43 (2.17-71.11) | 0.005 |  |  |
| Age, years | 1.02 | 0.337 |  |  |  |  |  |  |  |  |  |  |  |
| Male sex | 0.90 | 0.862 |  |  |  |  |  |  |  |  |  |  |  |
| BMI, kg/m^2^ | 1.05 | 0.355 |  |  |  |  |  |  |  |  |  |  |  |
| Hypertension | 0.69 | 0.513 |  |  |  |  |  |  |  |  |  |  |  |
| Diabetes mellitus | <0.001 | 0.998 |  |  |  |  |  |  |  |  |  |  |  |
| Hyperlipidemia | 0.83 | 0.810 |  |  |  |  |  |  |  |  |  |  |  |
| Coronary artery disease | 1.95 | 0.203 |  |  |  |  |  |  |  |  |  |  |  |
| Cerebrovascular disease | 1.76 | 0.591 |  |  |  |  |  |  |  |  |  |  |  |
| Smoke | 0.70 | 0.423 |  |  |  |  |  |  |  |  |  |  |  |
| Complicated TBAD | 0.55 | 0.179 |  |  |  |  |  |  |  |  |  |  |  |
| Extended to abdominal aorta | 1.07 | 0.903 |  |  |  |  |  |  |  |  |  |  |  |
| False lumen patency |  |  |  |  |  |  |  |  |  |  |  |  |  |
| Patent | Ref | Ref |  |  |  |  |  |  |  |  |  |  |  |
| Partial thrombosed | 0.76 | 0.592 |  |  |  |  |  |  |  |  |  |  |  |
| Completely thrombosed | <0.001 | 0.998 |  |  |  |  |  |  |  |  |  |  |  |
| Maximum aortic diameter in lesion, mm | 1.06 | 0.002 |  | 1.07 (1.02-1.11) | 0.003 |  | 1.06 (1.02-1.11) | 0.005 |  | 1.06 (1.02-1.11) | 0.007 |  |  |
| Pleural effusion | 0.67 | 0.372 |  |  |  |  |  |  |  |  |  |  |  |
| Pericardial effusion | 2.49 | 0.237 |  |  |  |  |  |  |  |  |  |  |  |
| WBC count, 10^9^/L | 1.12 | 0.011 |  |  |  |  |  |  |  |  |  |  |  |
| Monocyte count, 10^9^/L | 2.29 | 0.052 |  |  |  |  |  |  |  |  |  |  |  |
| Hemoglobin, g/L | 0.96 | <0.001 |  | 0.96 (0.94-0.98) | <0.001 |  | 0.95 (0.93-0.97) | <0.001 |  | 0.95 (0.93-0.98) | <0.001 |  |  |
| Platelet, 10^9^/L | 1.00 | 0.176 |  |  |  |  |  |  |  |  |  |  |  |
| Serum creatinine, mg/dL | 1.29 | 0.012 |  |  |  |  |  |  |  |  |  |  |  |
| D-Dimer, μg/ml | 1.08 | 0.016 |  |  |  |  |  |  |  |  |  |  |  |
| TC, mmol/L | 0.59 | 0.032 |  |  |  |  |  |  |  |  |  |  |  |
| TG, mmol/L | 0.75 | 0.444 |  |  |  |  |  |  |  |  |  |  |  |
| LDL-C, mmol/L | 0.56 | 0.051 |  |  |  |  |  |  |  |  |  |  |  |
| HDL-C, mmol/L | 0.18 | 0.046 |  |  |  |  |  |  |  |  |  |  |  |
| ALT, U/L | 1.01 | <0.001 |  |  |  |  |  |  |  |  |  |  |  |
| AST, U/L | 1.01 | <0.001 |  | 1.01 (1.01-1.02) | <0.001 |  | 1.02 (1.01-1.02) | <0.001 |  | 1.02 (1.01-1.02) | <0.001 |  |  |
| Antiplatelet drugs | 0.56 | 0.445 |  |  |  |  |  |  |  |  |  |  |  |
| ACEI | 1.89 | 0.180 |  |  |  |  |  |  |  |  |  |  |  |
| ARB | 0.36 | 0.048 |  |  |  |  |  |  |  |  |  |  |  |
| Beta-blockers | 0.64 | 0.561 |  |  |  |  |  |  |  |  |  |  |  |
| Calcium channel blockers | 0.41 | 0.050 |  | 0.28 (0.09-0.84) | 0.023 |  | 0.29 (0.10-0.84) | 0.023 |  | 0.28 (0.09-0.82) | 0.021 |  | |
| Statins | 0.77 | 0.573 |  |  |  |  |  |  |  |  |  |  |  |

ACEI indicates angiotensin-converting enzyme inhibitors; ALT, alanine aminotransferase; ARB, angiotensin receptor blockers; AST, aspartate aminotransferase; BMI, body mass index; CI, confidence interval; HDL-C, high density lipoprotein cholesterol; LDL-C, low density lipoprotein cholesterol; MHR, monocyte to high-density lipoprotein ratio; OR, odds ratio; TBAD, type B aortic dissection; TC, total cholesterol; TG, triglycerides; WBC, white blood cell.

**Supplementary Table 2. Details of cox regression analysis for long-term mortality before propensity score matching**

| **Variables** | **Univariate** | |  | **Multivariate** | | | | | | | |  |  |  |
| --- | --- | --- | --- | --- | --- | --- | --- | --- | --- | --- | --- | --- | --- | --- |
|  | **HR** | ***P*** |  | **Continuous MHR** | |  | **>1.13 vs** ≤**1.13** | |  | **Tertile** | |  |  |  |
|  |  |  |  | **HR (95% CI)** | ***P*** |  | **HR (95% CI)** | ***P*** |  | **HR (95% CI)** | ***P*** |  |  |  |
| Continuous MHR | 1.68 | <0.001 |  | 1.78 (1.31-2.41) | <0.001 |  |  |  |  |  |  |  |  |  |
| MHR, >1.13 vs ≤1.13 | 4.27 | <0.001 |  |  |  |  | 4.16 (2.13-8.10) | <0.001 |  |  |  |  |  |  |
| MHR |  |  |  |  |  |  |  |  |  |  |  |  |  |  |
| Tertile 1, <0.88 | Ref | Ref |  |  |  |  |  |  |  | Ref | Ref |  | | |
| Tertile 2, 0.88-1.33 | 1.81 | 0.203 |  |  |  |  |  |  |  | 1.71 (0.69-4.25) | 0.247 |  |  |  |
| Tertile 3, ≥1.33 | 3.94 | 0.001 |  |  |  |  |  |  |  | 3.78 (1.66-8.60) | 0.002 |  |  |  |
| Age, years | 1.02 | 0.177 |  |  |  |  |  |  |  |  |  |  |  |  |
| Male sex | 1.26 | 0.629 |  |  |  |  |  |  |  |  |  |  |  |  |
| BMI, kg/m^2^ | 0.94 | 0.132 |  |  |  |  |  |  |  |  |  |  |  |  |
| Hypertension | 0.89 | 0.763 |  |  |  |  |  |  |  |  |  |  |  |  |
| Diabetes mellitus | 1.06 | 0.925 |  |  |  |  |  |  |  |  |  |  |  |  |
| Hyperlipidemia | 0.78 | 0.631 |  |  |  |  |  |  |  |  |  |  |  |  |
| Coronary artery disease | 2.29 | 0.008 |  | 2.42 (1.30-4.51) | 0.005 |  | 2.07 (1.12-3.83) | 0.020 |  | 2.13 (1.15-3.94) | 0.016 |  |  |  |
| Cerebrovascular disease | 2.61 | 0.188 |  |  |  |  |  |  |  |  |  |  |  |  |
| Smoke | 0.77 | 0.340 |  |  |  |  |  |  |  |  |  |  |  |  |
| Complicated TBAD | 1.24 | 0.533 |  |  |  |  |  |  |  |  |  |  |  |  |
| Extended to abdominal aorta | 1.40 | 0.361 |  |  |  |  |  |  |  |  |  |  |  |  |
| False lumen patency |  |  |  |  |  |  |  |  |  |  |  |  |  |  |
| Patent | Ref | Ref |  |  |  |  |  |  |  |  |  |  |  |  |
| Partial thrombosed | 1.26 | 0.470 |  |  |  |  |  |  |  |  |  |  |  |  |
| Completely thrombosed | 0.56 | 0.559 |  |  |  |  |  |  |  |  |  |  |  |  |
| Maximum aortic diameter in lesion, mm | 1.03 | 0.028 |  | 1.03 (1.00-1.06) | 0.046 |  |  |  |  |  |  |  |  |  |
| Pleural effusion | 1.28 | 0.374 |  |  |  |  |  |  |  |  |  |  |  |  |
| Pericardial effusion | 1.53 | 0.474 |  |  |  |  |  |  |  |  |  |  |  |  |
| WBC count, 10^9^/L | 1.00 | 0.959 |  |  |  |  |  |  |  |  |  |  |  |  |
| Monocyte count, 10^9^/L | 1.77 | 0.022 |  |  |  |  |  |  |  |  |  |  |  |  |
| Hemoglobin, g/L | 0.99 | 0.123 |  |  |  |  |  |  |  |  |  |  |  |  |
| Platelet, 10^9^/L | 1.00 | 0.507 |  |  |  |  |  |  |  |  |  |  |  |  |
| Serum creatinine, mg/dL | 1.21 | 0.016 |  | 1.21 (1.02-1.44) | 0.029 |  | 1.23 (1.04-1.44) | 0.013 |  | 1.22 (1.05-1.43) | 0.012 |  |  |  |
| D-Dimer, μg/ml | 0.97 | 0.525 |  |  |  |  |  |  |  |  |  |  |  |  |
| TC, mmol/L | 0.85 | 0.287 |  |  |  |  |  |  |  |  |  |  |  |  |
| TG, mmol/L | 0.92 | 0.656 |  |  |  |  |  |  |  |  |  |  |  |  |
| LDL-C, mmol/L | 0.84 | 0.359 |  |  |  |  |  |  |  |  |  |  |  |  |
| HDL-C, mmol/L | 0.22 | 0.005 |  |  |  |  |  |  |  |  |  |  |  |  |
| ALT, U/L | 1.00 | 0.634 |  |  |  |  |  |  |  |  |  |  |  |  |
| AST, U/L | 1.01 | 0.069 |  |  |  |  |  |  |  |  |  |  |  |  |
| Antiplatelet drugs | 1.67 | 0.190 |  |  |  |  |  |  |  |  |  |  |  |  |
| ACEI | 0.54 | 0.076 |  |  |  |  |  |  |  |  |  |  |  |  |
| ARB | 1.35 | 0.288 |  |  |  |  |  |  |  |  |  |  |  |  |
| Beta-blockers | 1.06 | 0.924 |  |  |  |  |  |  |  |  |  |  |  |  |
| Calcium channel blockers | 1.26 | 0.550 |  |  |  |  |  |  |  |  |  |  | |  |
| Statins | 1.23 | 0.456 |  |  |  |  |  |  |  |  |  |  |  |  |

ACEI indicates angiotensin-converting enzyme inhibitors; ALT, alanine aminotransferase; ARB, angiotensin receptor blockers; AST, aspartate aminotransferase; BMI, body mass index; CI, confidence interval; HDL-C, high density lipoprotein cholesterol; HR, hazard ratio; LDL-C, low density lipoprotein cholesterol; MHR, monocyte to high-density lipoprotein ratio; TBAD, type B aortic dissection; TC, total cholesterol; TG, triglycerides; WBC, white blood cell.

**Supplementary Table 3. Details of logistic regression analysis for in-hospital mortality after propensity score matching**

| **Variables** | **Univariate** | |  | **Multivariate** | | | | |  |
| --- | --- | --- | --- | --- | --- | --- | --- | --- | --- |
|  | **OR** | ***P*** |  | **Continuous MHR** | |  | **>1.13 vs** ≤**1.13** | |  |
|  |  |  |  | **OR (95% CI)** | ***P*** |  | **OR (95% CI)** | ***P*** |  |
| Continuous MHR | 2.18 | 0.018 |  | 2.72 (1.23-5.99) | 0.013 |  |  |  |  |
| MHR, >1.13 vs ≤1.13 | 4.20 | 0.028 |  |  |  |  | 7.12 (1.52-33.45) | 0.013 |  |
| Age, years | 1.01 | 0.560 |  |  |  |  |  |  |  |
| Male sex | 0.35 | 0.115 |  |  |  |  |  |  |  |
| BMI, kg/m^2^ | 1.09 | 0.187 |  |  |  |  |  |  |  |
| Hypertension | 0.41 | 0.408 |  |  |  |  |  |  |  |
| Diabetes mellitus | <0.001 | 0.998 |  |  |  |  |  |  |  |
| Hyperlipidemia | 1.29 | 0.747 |  |  |  |  |  |  |  |
| Coronary artery disease | 0.83 | 0.811 |  |  |  |  |  |  |  |
| Cerebrovascular disease | <0.001 | 0.999 |  |  |  |  |  |  |  |
| Smoke | 0.68 | 0.470 |  |  |  |  |  |  |  |
| Complicated TBAD | 0.29 | 0.120 |  |  |  |  |  |  |  |
| Extended to abdominal aorta | 1.60 | 0.544 |  |  |  |  |  |  |  |
| False lumen patency |  |  |  |  |  |  |  |  |  |
| Patent | Ref | Ref |  |  |  |  |  |  |  |
| Partial thrombosed | 1.01 | 0.988 |  |  |  |  |  |  |  |
| Completely thrombosed | <0.001 | 0.999 |  |  |  |  |  |  |  |
| Maximum aortic diameter in lesion, mm | 1.05 | 0.018 |  | 1.06 (1.01-1.11) | 0.031 |  | 1.05 (1.00-1.11) | 0.048 |  |
| Pleural effusion | 0.56 | 0.291 |  |  |  |  |  |  |  |
| Pericardial effusion | 1.93 | 0.539 |  |  |  |  |  |  |  |
| WBC count, 10^9^/L | 1.09 | 0.104 |  |  |  |  |  |  |  |
| Monocyte count, 10^9^/L | 2.31 | 0.120 |  |  |  |  |  |  |  |
| Hemoglobin, g/L | 0.96 | <0.001 |  | 0.94 (0.92-0.97) | <0.001 |  | 0.94 (0.91-0.97) | <0.001 |  |
| Platelet, 10^9^/L | 1.00 | 0.524 |  |  |  |  |  |  |  |
| Serum creatinine, mg/dL | 1.29 | 0.032 |  |  |  |  |  |  |  |
| D-Dimer, μg/ml | 1.10 | 0.014 |  |  |  |  |  |  |  |
| TC, mmol/L | 0.49 | 0.028 |  |  |  |  |  |  |  |
| TG, mmol/L | 0.85 | 0.700 |  |  |  |  |  |  |  |
| LDL-C, mmol/L | 0.37 | 0.012 |  |  |  |  |  |  |  |
| HDL-C, mmol/L | 0.18 | 0.118 |  |  |  |  |  |  |  |
| ALT, U/L | 1.01 | 0.018 |  |  |  |  |  |  |  |
| AST, U/L | 1.01 | 0.001 |  | 1.02 (1.01-1.02) | <0.001 |  | 1.02 (1.01-1.02) | <0.001 |  |
| Antiplatelet drugs | 0.36 | 0.332 |  |  |  |  |  |  |  |
| ACEI | 1.00 | 1.0 |  |  |  |  |  |  |  |
| ARB | 0.43 | 0.151 |  |  |  |  |  |  |  |
| Beta-blockers | 0.46 | 0.321 |  |  |  |  |  |  |  |
| Calcium channel blockers | 0.74 | 0.607 |  |  |  |  |  |  |  |
| Statins | 0.86 | 0.785 |  |  |  |  |  |  |  |

ACEI indicates angiotensin-converting enzyme inhibitors; ALT, alanine aminotransferase; ARB, angiotensin receptor blockers; AST, aspartate aminotransferase; BMI, body mass index; CI, confidence interval; HDL-C, high density lipoprotein cholesterol; LDL-C, low density lipoprotein cholesterol; MHR, monocyte to high-density lipoprotein ratio; OR, odds ratio; TBAD, type B aortic dissection; TC, total cholesterol; TG, triglycerides; WBC, white blood cell.

**Supplementary Table 4. Details of cox regression analysis for long-term mortality after propensity score matching**

| **Variables** | **Univariate** | |  | **Multivariate** | | | | |  |
| --- | --- | --- | --- | --- | --- | --- | --- | --- | --- |
|  | **HR** | ***P*** |  | **Continuous MHR** | |  | **>1.13 vs** ≤**1.13** | |  |
|  |  |  |  | **HR (95% CI)** | ***P*** |  | **HR (95% CI)** | ***P*** |  |
| Continuous MHR | 1.77 | 0.003 |  | 1.87 (1.27-2.76) | 0.002 |  |  |  |  |
| MHR, >1.13 vs ≤1.13 | 3.89 | 0.001 |  |  |  |  | 4.02 (1.83-8.83) | 0.001 |  |
| Age, years | 1.03 | 0.060 |  | 1.03 (1.00-1.07) | 0.035 |  | 1.03 (1.00-1.06) | 0.039 |  |
| Male sex | 0.69 | 0.491 |  |  |  |  |  |  |  |
| BMI, kg/m^2^ | 0.93 | 0.146 |  |  |  |  |  |  |  |
| Hypertension | 0.68 | 0.360 |  |  |  |  |  |  |  |
| Diabetes mellitus | 1.19 | 0.808 |  |  |  |  |  |  |  |
| Hyperlipidemia | 1.11 | 0.839 |  |  |  |  |  |  |  |
| Coronary artery disease | 1.82 | 0.119 |  |  |  |  |  |  |  |
| Cerebrovascular disease | 3.34 | 0.100 |  |  |  |  |  |  |  |
| Smoke | 0.72 | 0.314 |  |  |  |  |  |  |  |
| Complicated TBAD | 1.13 | 0.756 |  |  |  |  |  |  |  |
| Extended to abdominal aorta | 1.34 | 0.507 |  |  |  |  |  |  |  |
| False lumen patency |  |  |  |  |  |  |  |  |  |
| Patent | Ref | Ref |  |  |  |  |  |  |  |
| Partial thrombosed | 0.84 | 0.688 |  |  |  |  |  |  |  |
| Completely thrombosed | <0.001 | 0.973 |  |  |  |  |  |  |  |
| Maximum aortic diameter in lesion, mm | 1.04 | 0.026 |  |  |  |  |  |  |  |
| Pleural effusion | 1.24 | 0.514 |  |  |  |  |  |  |  |
| Pericardial effusion | 0.05 | 0.482 |  |  |  |  |  |  |  |
| WBC count, 10^9^/L | 0.97 | 0.451 |  |  |  |  |  |  |  |
| Monocyte count, 10^9^/L | 1.96 | 0.052 |  |  |  |  |  |  |  |
| Hemoglobin, g/L | 0.99 | 0.169 |  |  |  |  |  |  |  |
| Platelet, 10^9^/L | 1.00 | 0.536 |  |  |  |  |  |  |  |
| Serum creatinine, mg/dL | 1.15 | 0.199 |  |  |  |  |  |  |  |
| D-Dimer, μg/ml | 1.00 | 0.996 |  |  |  |  |  |  |  |
| TC, mmol/L | 1.09 | 0.645 |  |  |  |  |  |  |  |
| TG, mmol/L | 1.11 | 0.645 |  |  |  |  |  |  |  |
| LDL-C, mmol/L | 1.08 | 0.741 |  |  |  |  |  |  |  |
| HDL-C, mmol/L | 0.25 | 0.039 |  |  |  |  |  |  |  |
| ALT, U/L | 1.00 | 0.631 |  |  |  |  |  |  |  |
| AST, U/L | 1.01 | 0.261 |  |  |  |  |  |  |  |
| Antiplatelet drugs | 2.10 | 0.066 |  |  |  |  |  |  |  |
| ACEI | 0.43 | 0.068 |  |  |  |  |  |  |  |
| ARB | 1.46 | 0.255 |  |  |  |  |  |  |  |
| Beta-blockers | 0.73 | 0.601 |  |  |  |  |  |  |  |
| Calcium channel blockers | 1.55 | 0.366 |  |  |  |  |  |  |  |
| Statins | 1.46 | 0.256 |  |  |  |  |  |  |  |

ACEI indicates angiotensin-converting enzyme inhibitors; ALT, alanine aminotransferase; ARB, angiotensin receptor blockers; AST, aspartate aminotransferase; BMI, body mass index; CI, confidence interval; HDL-C, high density lipoprotein cholesterol; HR, hazard ratio; LDL-C, low density lipoprotein cholesterol; MHR, monocyte to high-density lipoprotein ratio; TBAD, type B aortic dissection; TC, total cholesterol; TG, triglycerides; WBC, white blood cell.

**Supplementary Table 5. Details of cox regression analysis for long-term mortality in patients with age <65 years**

| **Variables** | **Univariate** | |  | **Multivariate** | | | | |  |
| --- | --- | --- | --- | --- | --- | --- | --- | --- | --- |
|  | **HR** | ***P*** |  | **Continuous MHR** | |  | **>1.13 vs** ≤**1.13** | |  |
|  |  |  |  | **HR (95% CI)** | ***P*** |  | **HR (95% CI)** | ***P*** |  |
| Continuous MHR | 1.54 | 0.015 |  | 1.61 (1.13-2.28) | 0.008 |  |  |  |  |
| MHR, >1.13 vs ≤1.13 | 3.55 | 0.001 |  |  |  |  | 3.92 (1.82-8.42) | <0.001 |  |
| Male sex | 1.72 | 0.366 |  |  |  |  |  |  |  |
| BMI, kg/m^2^ | 0.91 | 0.065 |  | 0.89 (0.80-0.99) | 0.029 |  | 0.89 (0.80-0.99) | 0.024 |  |
| Hypertension | 0.78 | 0.563 |  |  |  |  |  |  |  |
| Diabetes mellitus | 0.45 | 0.429 |  |  |  |  |  |  |  |
| Hyperlipidemia | 0.72 | 0.581 |  |  |  |  |  |  |  |
| Coronary artery disease | 1.40 | 0.453 |  |  |  |  |  |  |  |
| Cerebrovascular disease | 2.79 | 0.317 |  |  |  |  |  |  |  |
| Smoke | 0.76 | 0.394 |  |  |  |  |  |  |  |
| Complicated TBAD | 2.03 | 0.142 |  |  |  |  |  |  |  |
| Extended to abdominal aorta | 1.33 | 0.527 |  |  |  |  |  |  |  |
| False lumen patency |  |  |  |  |  |  |  |  |  |
| Patent | Ref | Ref |  |  |  |  |  |  |  |
| Partial thrombosed | 1.19 | 0.662 |  |  |  |  |  |  |  |
| Completely thrombosed | <0.001 | 0.975 |  |  |  |  |  |  |  |
| Maximum aortic diameter in lesion, mm | 1.02 | 0.311 |  |  |  |  |  |  |  |
| Pleural effusion | 1.42 | 0.287 |  |  |  |  |  |  |  |
| Pericardial effusion | 1.45 | 0.608 |  |  |  |  |  |  |  |
| WBC count, 10^9^/L | 0.99 | 0.804 |  |  |  |  |  |  |  |
| Monocyte count, 10^9^/L | 1.69 | 0.069 |  |  |  |  |  |  |  |
| Hemoglobin, g/L | 0.99 | 0.272 |  |  |  |  |  |  |  |
| Platelet, 10^9^/L | 1.00 | 0.848 |  |  |  |  |  |  |  |
| Serum creatinine, mg/dL | 1.22 | 0.028 |  | 1.24 (1.04-1.49) | 0.018 |  | 1.30 (1.07-1.57) | 0.007 |  |
| D-Dimer, μg/ml | 0.93 | 0.249 |  |  |  |  |  |  |  |
| TC, mmol/L | 0.82 | 0.268 |  |  |  |  |  |  |  |
| TG, mmol/L | 0.94 | 0.748 |  |  |  |  |  |  |  |
| LDL-C, mmol/L | 0.76 | 0.211 |  |  |  |  |  |  |  |
| HDL-C, mmol/L | 0.27 | 0.038 |  |  |  |  |  |  |  |
| ALT, U/L | 1.00 | 0.442 |  |  |  |  |  |  |  |
| AST, U/L | 1.01 | 0.206 |  |  |  |  |  |  |  |
| Antiplatelet drugs | 1.63 | 0.318 |  |  |  |  |  |  |  |
| ACEI | 0.44 | 0.058 |  |  |  |  |  |  |  |
| ARB | 1.33 | 0.388 |  |  |  |  |  |  |  |
| Beta-blockers | 0.78 | 0.672 |  |  |  |  |  |  |  |
| Calcium channel blockers | 1.15 | 0.762 |  |  |  |  |  |  |  |
| Statins | 1.63 | 0.140 |  |  |  |  |  |  |  |

ACEI indicates angiotensin-converting enzyme inhibitors; ALT, alanine aminotransferase; ARB, angiotensin receptor blockers; AST, aspartate aminotransferase; BMI, body mass index; CI, confidence interval; HDL-C, high density lipoprotein cholesterol; HR, hazard ratio; LDL-C, low density lipoprotein cholesterol; MHR, monocyte to high-density lipoprotein ratio; TBAD, type B aortic dissection; TC, total cholesterol; TG, triglycerides; WBC, white blood cell.

**Supplementary Table 6. Details of cox regression analysis for long-term mortality in patients with age ≥65 years**

| **Variables** | **Univariate** | |  | **Multivariate** | | | | |  |
| --- | --- | --- | --- | --- | --- | --- | --- | --- | --- |
|  | **HR** | ***P*** |  | **Continuous MHR** | |  | **>1.13 vs** ≤**1.13** | |  |
|  |  |  |  | **HR (95% CI)** | ***P*** |  | **HR (95% CI)** | ***P*** |  |
| Continuous MHR | 2.17 | 0.007 |  | 3.49 (1.66-7.35) | 0.001 |  |  |  |  |
| MHR, >1.13 vs ≤1.13 | 8.62 | 0.005 |  |  |  |  | 8.16 (1.78-37.42) | 0.007 |  |
| Male sex | 0.45 | 0.309 |  |  |  |  |  |  |  |
| BMI, kg/m^2^ | 1.04 | 0.592 |  |  |  |  |  |  |  |
| Hypertension | 1.43 | 0.729 |  |  |  |  |  |  |  |
| Diabetes mellitus | 5.24 | 0.034 |  |  |  |  |  |  |  |
| Hyperlipidemia | 1.16 | 0.884 |  |  |  |  |  |  |  |
| Coronary artery disease | 4.83 | 0.004 |  | 4.97 (1.47-16.86) | 0.010 |  | 4.45 (1.50-13.23) | 0.007 |  |
| Cerebrovascular disease | 1.92 | 0.535 |  |  |  |  |  |  |  |
| Smoke | 0.74 | 0.567 |  |  |  |  |  |  |  |
| Complicated TBAD | 0.54 | 0.261 |  |  |  |  |  |  |  |
| Extended to abdominal aorta | 1.80 | 0.368 |  |  |  |  |  |  |  |
| False lumen patency |  |  |  |  |  |  |  |  |  |
| Patent | Ref |  |  |  |  |  |  |  |  |
| Partial thrombosed | 1.17 | 0.782 |  |  |  |  |  |  |  |
| Completely thrombosed | 1.33 | 0.790 |  |  |  |  |  |  |  |
| Maximum aortic diameter in lesion, mm | 1.09 | 0.018 |  | 1.10 (1.01-1.19) | 0.031 |  |  |  |  |
| Pleural effusion | 0.85 | 0.763 |  |  |  |  |  |  |  |
| Pericardial effusion | 1.50 | 0.699 |  |  |  |  |  |  |  |
| WBC count, 10^9^/L | 1.07 | 0.328 |  |  |  |  |  |  |  |
| Monocyte count, 10^9^/L | 2.36 | 0.103 |  |  |  |  |  |  |  |
| Hemoglobin, g/L | 0.99 | 0.388 |  |  |  |  |  |  |  |
| Platelet, 10^9^/L | 1.00 | 0.128 |  |  |  |  |  |  |  |
| Serum creatinine, mg/dL | 1.26 | 0.199 |  |  |  |  |  |  |  |
| D-Dimer, μg/ml | 1.04 | 0.529 |  |  |  |  |  |  |  |
| TC, mmol/L | 1.00 | 0.989 |  |  |  |  |  |  |  |
| TG, mmol/L | 0.96 | 0.935 |  |  |  |  |  |  |  |
| LDL-C, mmol/L | 1.16 | 0.669 |  |  |  |  |  |  |  |
| HDL-C, mmol/L | 0.16 | 0.059 |  |  |  |  |  |  |  |
| ALT, U/L | 1.03 | 0.070 |  |  |  |  |  |  |  |
| AST, U/L | 1.03 | 0.025 |  |  |  |  |  |  |  |
| Antiplatelet drugs | 1.56 | 0.501 |  |  |  |  |  |  |  |
| ACEI | 0.79 | 0.691 |  |  |  |  |  |  |  |
| ARB | 1.41 | 0.520 |  |  |  |  |  |  |  |
| Beta-blockers | 21.94 | 0.598 |  |  |  |  |  |  |  |
| Calcium channel blockers | 1.58 | 0.560 |  |  |  |  |  |  |  |
| Statins | 0.55 | 0.282 |  |  |  |  |  |  |  |

ACEI indicates angiotensin-converting enzyme inhibitors; ALT, alanine aminotransferase; ARB, angiotensin receptor blockers; AST, aspartate aminotransferase; BMI, body mass index; CI, confidence interval; HDL-C, high density lipoprotein cholesterol; HR, hazard ratio; LDL-C, low density lipoprotein cholesterol; MHR, monocyte to high-density lipoprotein ratio; TBAD, type B aortic dissection; TC, total cholesterol; TG, triglycerides; WBC, white blood cell.

**Supplementary Table 7. Details of cox regression analysis for long-term mortality in male patients**

| **Variables** | **Univariate** | |  | **Multivariate** | | | | |  |
| --- | --- | --- | --- | --- | --- | --- | --- | --- | --- |
|  | **HR** | ***P*** |  | **Continuous MHR** | |  | **>1.13 vs** ≤**1.13** | |  |
|  |  |  |  | **HR (95% CI)** | ***P*** |  | **HR (95% CI)** | ***P*** |  |
| Continuous MHR | 1.60 | 0.003 |  | 1.69 (1.23-2.33) | 0.001 |  |  |  |  |
| MHR, >1.13 vs ≤1.13 | 3.37 | <0.001 |  |  |  |  | 3.53 (1.77-7.03) | <0.001 |  |
| Age, years | 1.01 | 0.330 |  |  |  |  |  |  |  |
| BMI, kg/m^2^ | 0.94 | 0.196 |  |  |  |  |  |  |  |
| Hypertension | 1.13 | 0.776 |  |  |  |  |  |  |  |
| Diabetes mellitus | 1.64 | 0.408 |  |  |  |  |  |  |  |
| Hyperlipidemia | 0.86 | 0.780 |  |  |  |  |  |  |  |
| Coronary artery disease | 2.40 | 0.007 |  | 2.70 (1.41-5.19) | 0.003 |  | 2.40 (1.26-4.57) | 0.008 |  |
| Cerebrovascular disease | 2.67 | 0.178 |  |  |  |  |  |  |  |
| Smoke | 0.72 | 0.256 |  |  |  |  |  |  |  |
| Complicated TBAD | 1.23 | 0.558 |  |  |  |  |  |  |  |
| Extended to abdominal aorta | 1.29 | 0.489 |  |  |  |  |  |  |  |
| False lumen patency |  |  |  |  |  |  |  |  |  |
| Patent | Ref | Ref |  |  |  |  |  |  |  |
| Partial thrombosed | 1.19 | 0.615 |  |  |  |  |  |  |  |
| Completely thrombosed | 0.55 | 0.552 |  |  |  |  |  |  |  |
| Maximum aortic diameter in lesion, mm | 1.03 | 0.060 |  |  |  |  |  |  |  |
| Pleural effusion | 1.21 | 0.514 |  |  |  |  |  |  |  |
| Pericardial effusion | 1.63 | 0.415 |  |  |  |  |  |  |  |
| WBC count, 10^9^/L | 1.00 | 0.990 |  |  |  |  |  |  |  |
| Monocyte count, 10^9^/L | 1.59 | 0.083 |  |  |  |  |  |  |  |
| Hemoglobin, g/L | 0.98 | 0.059 |  |  |  |  |  |  |  |
| Platelet, 10^9^/L | 1.00 | 0.779 |  |  |  |  |  |  |  |
| Serum creatinine, mg/dL | 1.22 | 0.014 |  | 1.26 (1.07-1.49) | 0.006 |  | 1.31 (1.10-1.55) | 0.002 |  |
| D-Dimer, μg/ml | 0.98 | 0.686 |  |  |  |  |  |  |  |
| TC, mmol/L | 0.84 | 0.286 |  |  |  |  |  |  |  |
| TG, mmol/L | 0.93 | 0.682 |  |  |  |  |  |  |  |
| LDL-C, mmol/L | 0.82 | 0.328 |  |  |  |  |  |  |  |
| HDL-C, mmol/L | 0.24 | 0.011 |  |  |  |  |  |  |  |
| ALT, U/L | 1.00 | 0.918 |  |  |  |  |  |  |  |
| AST, U/L | 1.01 | 0.019 |  |  |  |  |  |  |  |
| Antiplatelet drugs | 1.76 | 0.150 |  |  |  |  |  |  |  |
| ACEI | 0.50 | 0.065 |  |  |  |  |  |  |  |
| ARB | 1.45 | 0.209 |  |  |  |  |  |  |  |
| Beta-blockers | 0.99 | 0.981 |  |  |  |  |  |  |  |
| Calcium channel blockers | 1.08 | 0.841 |  |  |  |  |  |  |  |
| Statins | 1.25 | 0.450 |  |  |  |  |  |  |  |

ACEI indicates angiotensin-converting enzyme inhibitors; ALT, alanine aminotransferase; ARB, angiotensin receptor blockers; AST, aspartate aminotransferase; BMI, body mass index; CI, confidence interval; HDL-C; high density lipoprotein cholesterol; HR, hazard ratio; LDL-C, low density lipoprotein cholesterol; MHR, monocyte to high-density lipoprotein ratio; TBAD, type B aortic dissection; TC, total cholesterol; TG, triglycerides; WBC, white blood cell.

**Supplementary Table 8. Details of cox regression analysis for long-term mortality in female patients**

| **Variables** | **Univariate** | |  | **Multivariate** | | | | |  |
| --- | --- | --- | --- | --- | --- | --- | --- | --- | --- |
|  | **HR** | ***P*** |  | **Continuous MHR** | |  | **>1.13 vs** ≤**1.13** | |  |
|  |  |  |  | **HR (95% CI)** | ***P*** |  | **HR (95% CI)** | ***P*** |  |
| Continuous MHR | 7.34 | 0.010 |  | 17.16 (1.69-174.82) | 0.016 |  |  |  |  |
| MHR, >1.13 vs ≤1.13 | 235.84 | 0.222 |  |  |  |  | 235.84 (0.04-1.51E+6) | 0.222 |  |
| Age, years | 1.12 | 0.065 |  | 1.11 (1.01-1.21) | 0.032 |  |  |  |  |
| BMI, kg/m^2^ | 0.86 | 0.327 |  |  |  |  |  |  |  |
| Hypertension | 0.22 | 0.104 |  |  |  |  |  |  |  |
| Diabetes mellitus | 0.04 | 0.519 |  |  |  |  |  |  |  |
| Hyperlipidemia | 0.04 | 0.677 |  |  |  |  |  |  |  |
| Coronary artery disease | 1.38 | 0.774 |  |  |  |  |  |  |  |
| Cerebrovascular disease | 0.05 | 0.914 |  |  |  |  |  |  |  |
| Smoke | 0.05 | 0.833 |  |  |  |  |  |  |  |
| Complicated TBAD | 0.92 | 0.942 |  |  |  |  |  |  |  |
| Extended to abdominal aorta | 26.39 | 0.535 |  |  |  |  |  |  |  |
| False lumen patency |  |  |  |  |  |  |  |  |  |
| Patent | Ref | Ref |  |  |  |  |  |  |  |
| Partial thrombosed | 2.24 | 0.397 |  |  |  |  |  |  |  |
| Completely thrombosed | <0.001 | 0.995 |  |  |  |  |  |  |  |
| Maximum aortic diameter in lesion, mm | 1.16 | 0.060 |  |  |  |  |  |  |  |
| Pleural effusion | 2.97 | 0.332 |  |  |  |  |  |  |  |
| Pericardial effusion | 0.05 | 0.825 |  |  |  |  |  |  |  |
| WBC count, 10^9^/L | 1.03 | 0.823 |  |  |  |  |  |  |  |
| Monocyte count, 10^9^/L | 10.07 | 0.037 |  |  |  |  |  |  |  |
| Hemoglobin, g/L | 1.00 | 0.881 |  |  |  |  |  |  |  |
| Platelet, 10^9^/L | 1.01 | 0.063 |  |  |  |  |  |  |  |
| Serum creatinine, mg/dL | 1.03 | 0.961 |  |  |  |  |  |  |  |
| D-Dimer, μg/ml | 0.56 | 0.236 |  |  |  |  |  |  |  |
| TC, mmol/L | 1.11 | 0.827 |  |  |  |  |  |  |  |
| TG, mmol/L | 1.02 | 0.981 |  |  |  |  |  |  |  |
| LDL-C, mmol/L | 1.19 | 0.749 |  |  |  |  |  |  |  |
| HDL-C, mmol/L | 0.12 | 0.220 |  |  |  |  |  |  |  |
| ALT, U/L | 0.93 | 0.257 |  |  |  |  |  |  |  |
| AST, U/L | 0.98 | 0.546 |  |  |  |  |  |  |  |
| Antiplatelet drugs | 0.04 | 0.761 |  |  |  |  |  |  |  |
| ACEI | 0.88 | 0.903 |  |  |  |  |  |  |  |
| ARB | 0.66 | 0.722 |  |  |  |  |  |  |  |
| Beta-blockers | 22.12 | 0.812 |  |  |  |  |  |  |  |
| Calcium channel blockers | 28.95 | 0.485 |  |  |  |  |  |  |  |
| Statins | 1.18 | 0.854 |  |  |  |  |  |  |  |

ACEI indicates angiotensin-converting enzyme inhibitors; ALT, alanine aminotransferase; ARB, angiotensin receptor blockers; AST, aspartate aminotransferase; BMI, body mass index; CI, confidence interval; HDL-C; high density lipoprotein cholesterol; HR, hazard ratio; LDL-C, low density lipoprotein cholesterol; MHR, monocyte to high-density lipoprotein ratio; TBAD, type B aortic dissection; TC, total cholesterol; TG, triglycerides; WBC, white blood cell.

**Supplementary Table 9. Details of cox regression analysis for long-term mortality in patients with uncomplicated aortic dissection**

| **Variables** | **Univariate** | |  | **Multivariate** | | | | |  |
| --- | --- | --- | --- | --- | --- | --- | --- | --- | --- |
|  | **HR** | ***P*** |  | **Continuous MHR** | |  | **>1.13 vs** ≤**1.13** | |  |
|  |  |  |  | **HR (95% CI)** | ***P*** |  | **HR (95% CI)** | ***P*** |  |
| Continuous MHR | 2.64 | 0.017 |  | 3.06 (1.31-7.20) | 0.010 |  |  |  |  |
| MHR, >1.13 vs ≤1.13 | 4.79 | 0.021 |  |  |  |  | 4.79 (1.26-18.17) | 0.021 |  |
| Age, years | 1.03 | 0.386 |  |  |  |  |  |  |  |
| Male sex | 1.61 | 0.653 |  |  |  |  |  |  |  |
| BMI, kg/m^2^ | 1.01 | 0.958 |  |  |  |  |  |  |  |
| Hypertension | 26.73 | 0.347 |  |  |  |  |  |  |  |
| Diabetes mellitus | 0.05 | 0.618 |  |  |  |  |  |  |  |
| Hyperlipidemia | 0.71 | 0.745 |  |  |  |  |  |  |  |
| Coronary artery disease | 2.96 | 0.084 |  | 3.72 (1.04-13.35) | 0.044 |  |  |  |  |
| Cerebrovascular disease | 0.05 | 0.652 |  |  |  |  |  |  |  |
| Smoke | 2.01 | 0.266 |  |  |  |  |  |  |  |
| Extended to abdominal aorta | 2.47 | 0.391 |  |  |  |  |  |  |  |
| False lumen patency |  |  |  |  |  |  |  |  |  |
| Patent | Ref | Ref |  |  |  |  |  |  |  |
| Partial thrombosed | 1.10 | 0.894 |  |  |  |  |  |  |  |
| Completely thrombosed | <0.001 | 0.985 |  |  |  |  |  |  |  |
| Maximum aortic diameter in lesion, mm | 1.01 | 0.694 |  |  |  |  |  |  |  |
| Pleural effusion | 1.26 | 0.706 |  |  |  |  |  |  |  |
| Pericardial effusion | 0.05 | 0.779 |  |  |  |  |  |  |  |
| WBC count, 10^9^/L | 1.12 | 0.121 |  |  |  |  |  |  |  |
| Monocyte count, 10^9^/L | 6.30 | 0.019 |  |  |  |  |  |  |  |
| Hemoglobin, g/L | 0.99 | 0.481 |  |  |  |  |  |  |  |
| Platelet, 10^9^/L | 1.00 | 0.670 |  |  |  |  |  |  |  |
| Serum creatinine, mg/dL | 1.18 | 0.210 |  |  |  |  |  |  |  |
| D-Dimer, μg/ml | 0.93 | 0.475 |  |  |  |  |  |  |  |
| TC, mmol/L | 0.95 | 0.871 |  |  |  |  |  |  |  |
| TG, mmol/L | 0.60 | 0.368 |  |  |  |  |  |  |  |
| LDL-C, mmol/L | 0.90 | 0.790 |  |  |  |  |  |  |  |
| HDL-C, mmol/L | 0.43 | 0.512 |  |  |  |  |  |  |  |
| ALT, U/L | 0.99 | 0.580 |  |  |  |  |  |  |  |
| AST, U/L | 1.00 | 0.699 |  |  |  |  |  |  |  |
| Antiplatelet drugs | 1.16 | 0.850 |  |  |  |  |  |  |  |
| ACEI | 1.30 | 0.706 |  |  |  |  |  |  |  |
| ARB | 0.89 | 0.859 |  |  |  |  |  |  |  |
| Beta-blockers | 0.44 | 0.440 |  |  |  |  |  |  |  |
| Calcium channel blockers | 1.52 | 0.597 |  |  |  |  |  |  |  |
| Statins | 0.91 | 0.878 |  |  |  |  |  |  |  |

ACEI indicates angiotensin-converting enzyme inhibitors; ALT, alanine aminotransferase; ARB, angiotensin receptor blockers; AST, aspartate aminotransferase; BMI, body mass index; CI, confidence interval; HDL-C, high density lipoprotein cholesterol; HR, hazard ratio; LDL-C, low density lipoprotein cholesterol; MHR, monocyte to high-density lipoprotein ratio; TC, total cholesterol; TG, triglycerides; WBC, white blood cell.

**Supplementary Table 10. Details of cox regression analysis for long-term mortality in patients with complicated aortic dissection**

| **Variables** | **Univariate** | |  | **Multivariate** | | | | |  |
| --- | --- | --- | --- | --- | --- | --- | --- | --- | --- |
|  | **HR** | ***P*** |  | **Continuous MHR** | |  | **>1.13 vs** ≤**1.13** | |  |
|  |  |  |  | **HR (95% CI)** | ***P*** |  | **HR (95% CI)** | ***P*** |  |
| Continuous MHR | 1.56 | 0.007 |  | 1.34 (0.87-2.06) | 0.185 |  |  |  |  |
| MHR, >1.13 vs ≤1.13 | 4.18 | <0.001 |  |  |  |  | 4.64 (2.12-10.18) | <0.001 |  |
| Age, years | 1.02 | 0.259 |  |  |  |  |  |  |  |
| Male sex | 1.23 | 0.696 |  |  |  |  |  |  |  |
| BMI, kg/m^2^ | 0.92 | 0.083 |  | 0.91 (0.83-1.01) | 0.067 |  | 0.88 (0.80-0.98) | 0.014 |  |
| Hypertension | 0.64 | 0.253 |  |  |  |  |  |  |  |
| Diabetes mellitus | 1.32 | 0.644 |  |  |  |  |  |  |  |
| Hyperlipidemia | 0.80 | 0.711 |  |  |  |  |  |  |  |
| Coronary artery disease | 2.11 | 0.040 |  | 2.13 (0.99-4.56) | 0.052 |  |  |  |  |
| Cerebrovascular disease | 6.47 | 0.011 |  | 3.09 (0.53-18.12) | 0.211 |  | 4.08 (0.91-18.29) | 0.066 |  |
| Smoke | 0.58 | 0.090 |  | 0.63 (0.32-1.22) | 0.170 |  |  |  |  |
| Extended to abdominal aorta | 1.24 | 0.583 |  |  |  |  |  |  |  |
| False lumen patency |  |  |  |  |  |  |  |  |  |
| Patent | Ref | Ref |  |  |  |  |  |  |  |
| Partial thrombosed | 1.28 | 0.486 |  |  |  |  |  |  |  |
| Completely thrombosed | 0.58 | 0.592 |  |  |  |  |  |  |  |
| Maximum aortic diameter in lesion, mm | 1.05 | 0.005 |  | 1.04 (1.00-1.08) | 0.041 |  | 1.04 (1.01-1.08) | 0.021 |  |
| Pleural effusion | 1.27 | 0.448 |  |  |  |  |  |  |  |
| Pericardial effusion | 1.68 | 0.387 |  |  |  |  |  |  |  |
| WBC count, 10^9^/L | 0.96 | 0.373 |  |  |  |  |  |  |  |
| Monocyte count, 10^9^/L | 1.47 | 0.177 |  |  |  |  |  |  |  |
| Hemoglobin, g/L | 0.99 | 0.163 |  |  |  |  |  |  |  |
| Platelet, 10^9^/L | 1.00 | 0.466 |  |  |  |  |  |  |  |
| Serum creatinine, mg/dL | 1.26 | 0.022 |  | 1.25 (0.98-1.60) | 0.072 |  | 1.32 (1.04-1.68) | 0.022 |  |
| D-Dimer, μg/ml | 0.99 | 0.810 |  |  |  |  |  |  |  |
| TC, mmol/L | 0.82 | 0.245 |  |  |  |  |  |  |  |
| TG, mmol/L | 0.97 | 0.873 |  |  |  |  |  |  |  |
| LDL-C, mmol/L | 0.83 | 0.383 |  |  |  |  |  |  |  |
| HDL-C, mmol/L | 0.17 | 0.003 |  | 0.38 (0.09-1.59) | 0.183 |  |  |  |  |
| ALT, U/L | 1.00 | 0.903 |  |  |  |  |  |  |  |
| AST, U/L | 1.01 | 0.038 |  | 1.00 (0.99-1.01) | 0.792 |  |  |  |  |
| Antiplatelet drugs | 1.90 | 0.153 |  |  |  |  |  |  |  |
| ACEI | 0.42 | 0.033 |  | 0.52 (0.23-1.16) | 0.109 |  |  |  |  |
| ARB | 1.46 | 0.230 |  |  |  |  |  |  |  |
| Beta-blockers | 1.42 | 0.633 |  |  |  |  |  |  |  |
| Calcium channel blockers | 1.13 | 0.786 |  |  |  |  |  |  |  |
| Statins | 1.40 | 0.288 |  |  |  |  |  |  |  |

ACEI indicates angiotensin-converting enzyme inhibitors; ALT, alanine aminotransferase; ARB, angiotensin receptor blockers; AST, aspartate aminotransferase; BMI, body mass index; CI, confidence interval; HDL-C, high density lipoprotein cholesterol; HR, hazard ratio; LDL-C, low density lipoprotein cholesterol; MHR, monocyte to high-density lipoprotein ratio; TC, total cholesterol; TG, triglycerides; WBC, white blood cell.

**Supplementary Table 11. Details of cox regression analysis for long-term mortality in patients without anemia**

| **Variables** | **Univariate** | |  | **Multivariate** | | | | |  |
| --- | --- | --- | --- | --- | --- | --- | --- | --- | --- |
|  | **HR** | ***P*** |  | **Continuous MHR** | |  | **>1.13 vs** ≤**1.13** | |  |
|  |  |  |  | **HR (95% CI)** | ***P*** |  | **HR (95% CI)** | ***P*** |  |
| Continuous MHR | 2.01 | 0.006 |  | 2.25 (1.33-3.82) | 0.003 |  |  |  |  |
| MHR, >1.13 vs ≤1.13 | 6.03 | 0.004 |  |  |  |  | 5.83 (1.70-20.06) | 0.005 |  |
| Age, years | 1.00 | 0.960 |  |  |  |  |  |  |  |
| Male sex | 1.05 | 0.945 |  |  |  |  |  |  |  |
| BMI, kg/m^2^ | 1.05 | 0.427 |  |  |  |  |  |  |  |
| Hypertension | 0.59 | 0.315 |  |  |  |  |  |  |  |
| Diabetes mellitus | 1.00 | 0.999 |  |  |  |  |  |  |  |
| Hyperlipidemia | 0.57 | 0.587 |  |  |  |  |  |  |  |
| Coronary artery disease | 0.82 | 0.792 |  |  |  |  |  |  |  |
| Cerebrovascular disease | 0.05 | 0.748 |  |  |  |  |  |  |  |
| Smoke | 0.96 | 0.930 |  |  |  |  |  |  |  |
| Complicated TBAD | 1.31 | 0.630 |  |  |  |  |  |  |  |
| Extended to abdominal aorta | 1.07 | 0.908 |  |  |  |  |  |  |  |
| False lumen patency |  |  |  |  |  |  |  |  |  |
| Patent | Ref | Ref |  |  |  |  |  |  |  |
| Partial thrombosed | 1.08 | 0.895 |  |  |  |  |  |  |  |
| Completely thrombosed | 1.17 | 0.879 |  |  |  |  |  |  |  |
| Maximum aortic diameter in lesion, mm | 1.06 | 0.022 |  | 1.06 (1.02-1.12) | 0.009 |  | 1.05 (1.00-1.11) | 0.041 |  |
| Pleural effusion | 1.56 | 0.342 |  |  |  |  |  |  |  |
| Pericardial effusion | 0.05 | 0.784 |  |  |  |  |  |  |  |
| WBC count, 10^9^/L | 1.06 | 0.266 |  |  |  |  |  |  |  |
| Monocyte count, 10^9^/L | 2.43 | 0.034 |  |  |  |  |  |  |  |
| Platelet, 10^9^/L | 1.00 | 0.539 |  |  |  |  |  |  |  |
| Serum creatinine, mg/dL | 1.38 | 0.254 |  |  |  |  |  |  |  |
| D-Dimer, μg/ml | 0.99 | 0.873 |  |  |  |  |  |  |  |
| TC, mmol/L | 1.15 | 0.486 |  |  |  |  |  |  |  |
| TG, mmol/L | 1.05 | 0.762 |  |  |  |  |  |  |  |
| LDL-C, mmol/L | 1.15 | 0.668 |  |  |  |  |  |  |  |
| HDL-C, mmol/L | 0.34 | 0.227 |  |  |  |  |  |  |  |
| ALT, U/L | 0.99 | 0.338 |  |  |  |  |  |  |  |
| AST, U/L | 0.99 | 0.525 |  |  |  |  |  |  |  |
| Antiplatelet drugs | 0.42 | 0.399 |  |  |  |  |  |  |  |
| ACEI | 0.70 | 0.519 |  |  |  |  |  |  |  |
| ARB | 1.32 | 0.556 |  |  |  |  |  |  |  |
| Beta-blockers | 0.84 | 0.862 |  |  |  |  |  |  |  |
| Calcium channel blockers | 1.14 | 0.838 |  |  |  |  |  |  |  |
| Statins | 1.16 | 0.743 |  |  |  |  |  |  |  |

ACEI indicates angiotensin-converting enzyme inhibitors; ALT, alanine aminotransferase; ARB, angiotensin receptor blockers; AST, aspartate aminotransferase; BMI, body mass index; CI, confidence interval; HDL-C, high density lipoprotein cholesterol; HR, hazard ratio; LDL-C, low density lipoprotein cholesterol; MHR, monocyte to high-density lipoprotein ratio; TBAD, type B aortic dissection; TC, total cholesterol; TG, triglycerides; WBC, white blood cell.

**Supplementary Table 12. Details of cox regression analysis for long-term mortality in patients with anemia**

| **Variables** | **Univariate** | |  | **Multivariate** | | | | |  |
| --- | --- | --- | --- | --- | --- | --- | --- | --- | --- |
|  | **HR** | ***P*** |  | **Continuous MHR** | |  | **>1.13 vs** ≤**1.13** | |  |
|  |  |  |  | **HR (95% CI)** | ***P*** |  | **HR (95% CI)** | ***P*** |  |
| Continuous MHR | 1.45 | 0.043 |  | 1.43 (0.86-2.37) | 0.165 |  |  |  |  |
| MHR, >1.13 vs ≤1.13 | 3.51 | 0.002 |  |  |  |  | 3.66 (1.60-8.38) | 0.002 |  |
| Age, years | 1.02 | 0.234 |  |  |  |  |  |  |  |
| Male sex | 1.49 | 0.515 |  |  |  |  |  |  |  |
| BMI, kg/m^2^ | 0.90 | 0.047 |  | 0.89 (0.79-1.07) | 0.062 |  |  |  |  |
| Hypertension | 1.08 | 0.901 |  |  |  |  |  |  |  |
| Diabetes mellitus | 0.98 | 0.976 |  |  |  |  |  |  |  |
| Hyperlipidemia | 0.84 | 0.775 |  |  |  |  |  |  |  |
| Coronary artery disease | 3.18 | 0.002 |  | 2.38 (1.02-5.58) | 0.045 |  | 2.37 (1.04-5.38) | 0.040 |  |
| Cerebrovascular disease | 3.01 | 0.136 |  |  |  |  |  |  |  |
| Smoke | 0.69 | 0.302 |  |  |  |  |  |  |  |
| Complicated TBAD | 1.20 | 0.668 |  |  |  |  |  |  |  |
| Extended to abdominal aorta | 1.54 | 0.372 |  |  |  |  |  |  |  |
| False lumen patency |  |  |  |  |  |  |  |  |  |
| Patent | Ref | Ref |  |  |  |  |  |  |  |
| Partial thrombosed | 1.19 | 0.654 |  |  |  |  |  |  |  |
| Completely thrombosed | <0.001 | 0.979 |  |  |  |  |  |  |  |
| Maximum aortic diameter in lesion, mm | 1.02 | 0.253 |  |  |  |  |  |  |  |
| Pleural effusion | 1.13 | 0.719 |  |  |  |  |  |  |  |
| Pericardial effusion | 1.25 | 0.715 |  |  |  |  |  |  |  |
| WBC count, 10^9^/L | 0.97 | 0.571 |  |  |  |  |  |  |  |
| Monocyte count, 10^9^/L | 1.51 | 0.190 |  |  |  |  |  |  |  |
| Platelet, 10^9^/L | 1.00 | 0.299 |  |  |  |  |  |  |  |
| Serum creatinine, mg/dL | 1.16 | 0.094 |  | 1.18 (0.95-1.48) | 0.132 |  |  |  |  |
| D-Dimer, μg/ml | 0.96 | 0.511 |  |  |  |  |  |  |  |
| TC, mmol/L | 0.78 | 0.202 |  |  |  |  |  |  |  |
| TG, mmol/L | 0.81 | 0.447 |  |  |  |  |  |  |  |
| LDL-C, mmol/L | 0.85 | 0.493 |  |  |  |  |  |  |  |
| HDL-C, mmol/L | 0.22 | 0.021 |  | 0.50 (0.11-2.26) | 0.367 |  |  |  |  |
| ALT, U/L | 1.00 | 0.935 |  |  |  |  |  |  |  |
| AST, U/L | 1.01 | 0.015 |  | 1.01 (1.00-1.02) | 0.011 |  | 1.01 (1.00-1.02) | 0.026 |  |
| Antiplatelet drugs | 2.74 | 0.020 |  | 1.88 (0.65-5.41) | 0.244 |  | 2.71 (0.99-7.41) | 0.052 |  |
| ACEI | 0.54 | 0.178 |  |  |  |  |  |  |  |
| ARB | 1.30 | 0.459 |  |  |  |  |  |  |  |
| Beta-blockers | 1.44 | 0.617 |  |  |  |  |  |  |  |
| Calcium channel blockers | 1.43 | 0.462 |  |  |  |  |  |  |  |
| Statins | 1.36 | 0.383 |  |  |  |  |  |  |  |

ACEI indicates angiotensin-converting enzyme inhibitors; ALT, alanine aminotransferase; ARB, angiotensin receptor blockers; AST, aspartate aminotransferase; BMI, body mass index; CI, confidence interval; HDL-C, high density lipoprotein cholesterol; HR, hazard ratio; LDL-C, low density lipoprotein cholesterol; MHR, monocyte to high-density lipoprotein ratio; TBAD, type B aortic dissection; TC, total cholesterol; TG, triglycerides; WBC, white blood cell.

**Supplementary Table 13. Details of cox regression analysis for long-term mortality in patients with eGFR <60 mL/min/1.73 m^2^**

| **Variables** | **Univariate** | |  | **Multivariate** | | | | |  |
| --- | --- | --- | --- | --- | --- | --- | --- | --- | --- |
|  | **HR** | ***P*** |  | **Continuous MHR** | |  | **>1.13 vs** ≤**1.13** | |  |
|  |  |  |  | **HR (95% CI)** | ***P*** |  | **HR (95% CI)** | ***P*** |  |
| Continuous MHR | 1.64 | 0.006 |  | 1.79 (1.22-2.61) | 0.003 |  |  |  |  |
| MHR, >1.13 vs ≤1.13 | 5.60 | 0.002 |  |  |  |  | 8.91 (2.82-28.16) | <0.001 |  |
| Age, years | 1.02 | 0.192 |  |  |  |  |  |  |  |
| Male sex | 1.17 | 0.877 |  |  |  |  |  |  |  |
| BMI, kg/m^2^ | 0.98 | 0.717 |  |  |  |  |  |  |  |
| Hypertension | 1.64 | 0.629 |  |  |  |  |  |  |  |
| Diabetes mellitus | 1.87 | 0.316 |  |  |  |  |  |  |  |
| Hyperlipidemia | 0.63 | 0.459 |  |  |  |  |  |  |  |
| Coronary artery disease | 1.67 | 0.258 |  |  |  |  |  |  |  |
| Cerebrovascular disease | 2.60 | 0.204 |  |  |  |  |  |  |  |
| Smoke | 0.74 | 0.471 |  |  |  |  |  |  |  |
| Complicated TBAD | 1.25 | 0.634 |  |  |  |  |  |  |  |
| Extended to abdominal aorta | 1.57 | 0.408 |  |  |  |  |  |  |  |
| False lumen patency |  |  |  |  |  |  |  |  |  |
| Patent | Ref | Ref |  |  |  |  |  |  |  |
| Partial thrombosed | 1.02 | 0.965 |  |  |  |  |  |  |  |
| Completely thrombosed | 2.55 | 0.371 |  |  |  |  |  |  |  |
| Maximum aortic diameter in lesion, mm | 1.04 | 0.065 |  |  |  |  |  |  |  |
| Pleural effusion | 1.47 | 0.351 |  |  |  |  |  |  |  |
| Pericardial effusion | 1.91 | 0.298 |  |  |  |  |  |  |  |
| WBC count, 10^9^/L | 1.02 | 0.579 |  |  |  |  |  |  |  |
| Monocyte count, 10^9^/L | 2.32 | 0.012 |  |  |  |  |  |  |  |
| Hemoglobin, g/L | 0.99 | 0.180 |  |  |  |  |  |  |  |
| Platelet, 10^9^/L | 1.00 | 0.460 |  |  |  |  |  |  |  |
| Serum creatinine, mg/dL | 1.09 | 0.473 |  |  |  |  |  |  |  |
| D-Dimer, μg/ml | 0.96 | 0.493 |  |  |  |  |  |  |  |
| TC, mmol/L | 0.80 | 0.319 |  |  |  |  |  |  |  |
| TG, mmol/L | 0.77 | 0.375 |  |  |  |  |  |  |  |
| LDL-C, mmol/L | 0.93 | 0.780 |  |  |  |  |  |  |  |
| HDL-C, mmol/L | 0.21 | 0.044 |  |  |  |  |  |  |  |
| ALT, U/L | 1.00 | 0.671 |  |  |  |  |  |  |  |
| AST, U/L | 1.01 | 0.050 |  | 1.01 (1.00-1.02) | 0.041 |  |  |  |  |
| Antiplatelet drugs | 2.21 | 0.085 |  | 3.57 (1.34-9.49) | 0.011 |  | 4.95 (1.82-13.50) |  |  |
| ACEI | 0.40 | 0.115 |  |  |  |  |  |  |  |
| ARB | 0.98 | 0.961 |  |  |  |  |  |  |  |
| Beta-blockers | 0.87 | 0.845 |  |  |  |  |  |  |  |
| Calcium channel blockers | 2.52 | 0.212 |  |  |  |  |  |  |  |
| Statins | 1.60 | 0.264 |  |  |  |  |  |  |  |

ACEI indicates angiotensin-converting enzyme inhibitors; ALT, alanine aminotransferase; ARB, angiotensin receptor blockers; AST, aspartate aminotransferase; BMI, body mass index; CI, confidence interval; HDL-C; eGFR, estimated glomerular filtration rate; high density lipoprotein cholesterol; HR, hazard ratio; LDL-C, low density lipoprotein cholesterol; MHR, monocyte to high-density lipoprotein ratio; TBAD, type B aortic dissection; TC, total cholesterol; TG, triglycerides; WBC, white blood cell.

**Supplementary Table 14. Details of cox regression analysis for long-term mortality in patients with eGFR ≥60 mL/min/1.73 m^2^**

| **Variables** | **Univariate** | |  | **Multivariate** | | | | |  |
| --- | --- | --- | --- | --- | --- | --- | --- | --- | --- |
|  | **HR** | ***P*** |  | **Continuous MHR** | |  | **>1.13 vs** ≤**1.13** | |  |
|  |  |  |  | **HR (95% CI)** | ***P*** |  | **HR (95% CI)** | ***P*** |  |
| Continuous MHR | 1.69 | 0.038 |  | 1.76 (1.08-2.88) | 0.025 |  |  |  |  |
| MHR, >1.13 vs ≤1.13 | 3.46 | 0.004 |  |  |  |  | 3.59 (1.53-8.47) | 0.003 |  |
| Age, years | 1.01 | 0.624 |  |  |  |  |  |  |  |
| Male sex | 1.00 | 1.0 |  |  |  |  |  |  |  |
| BMI, kg/m^2^ | 0.90 | 0.061 |  | 0.89 (0.79-1.00) | 0.044 |  | 0.88 (0.78-1.00) | 0.045 |  |
| Hypertension | 0.64 | 0.301 |  |  |  |  |  |  |  |
| Diabetes mellitus | 0.05 | 0.414 |  |  |  |  |  |  |  |
| Hyperlipidemia | 0.54 | 0.544 |  |  |  |  |  |  |  |
| Coronary artery disease | 2.37 | 0.049 |  |  |  |  |  |  |  |
| Cerebrovascular disease | 0.05 | 0.763 |  |  |  |  |  |  |  |
| Smoke | 0.67 | 0.476 |  |  |  |  |  |  |  |
| Complicated TBAD | 1.40 | 0.499 |  |  |  |  |  |  |  |
| Extended to abdominal aorta | 1.37 | 0.522 |  |  |  |  |  |  |  |
| False lumen patency |  |  |  |  |  |  |  |  |  |
| Patent | Ref | Ref |  |  |  |  |  |  |  |
| Partial thrombosed | 1.36 | 0.470 |  |  |  |  |  |  |  |
| Completely thrombosed | <0.001 | 0.975 |  |  |  |  |  |  |  |
| Maximum aortic diameter in lesion, mm | 1.01 | 0.637 |  |  |  |  |  |  |  |
| Pleural effusion | 1.20 | 0.631 |  |  |  |  |  |  |  |
| Pericardial effusion | 0.05 | 0.557 |  |  |  |  |  |  |  |
| WBC count, 10^9^/L | 0.94 | 0.323 |  |  |  |  |  |  |  |
| Monocyte count, 10^9^/L | 1.57 | 0.224 |  |  |  |  |  |  |  |
| Hemoglobin, g/L | 1.00 | 0.937 |  |  |  |  |  |  |  |
| Platelet, 10^9^/L | 1.00 | 0.262 |  |  |  |  |  |  |  |
| Serum creatinine, mg/dL | 1.18 | 0.859 |  |  |  |  |  |  |  |
| D-Dimer, μg/ml | 0.97 | 0.642 |  |  |  |  |  |  |  |
| TC, mmol/L | 0.93 | 0.710 |  |  |  |  |  |  |  |
| TG, mmol/L | 0.88 | 0.667 |  |  |  |  |  |  |  |
| LDL-C, mmol/L | 0.89 | 0.637 |  |  |  |  |  |  |  |
| HDL-C, mmol/L | 0.31 | 0.100 |  |  |  |  |  |  |  |
| ALT, U/L | 1.00 | 0.542 |  |  |  |  |  |  |  |
| AST, U/L | 1.00 | 0.853 |  |  |  |  |  |  |  |
| Antiplatelet drugs | 0.45 | 0.435 |  |  |  |  |  |  |  |
| ACEI | 0.68 | 0.388 |  |  |  |  |  |  |  |
| ARB | 1.78 | 0.133 |  |  |  |  |  |  |  |
| Beta-blockers | 1.67 | 0.615 |  |  |  |  |  |  |  |
| Calcium channel blockers | 0.83 | 0.683 |  |  |  |  |  |  |  |
| Statins | 0.95 | 0.901 |  |  |  |  |  |  |  |

ACEI indicates angiotensin-converting enzyme inhibitors; ALT, alanine aminotransferase; ARB, angiotensin receptor blockers; AST, aspartate aminotransferase; BMI, body mass index; CI, confidence interval; HDL-C; eGFR, estimated glomerular filtration rate; high density lipoprotein cholesterol; HR, hazard ratio; LDL-C, low density lipoprotein cholesterol; MHR, monocyte to high-density lipoprotein ratio; TBAD, type B aortic dissection; TC, total cholesterol; TG, triglycerides; WBC, white blood cell.
