# Supplementary figures and images for: Association Between Preoperative Monocyte to High-Density Lipoprotein Ratio on In-hospital and Long-Term Mortality in Patients Undergoing Endovascular Repair for Acute Type B Aortic Dissection
Source: Front Cardiovasc Med. 2022 Jan 7;8:775471. doi: 10.3389/fcvm.2021.775471 (PMC8777016; doi:10.3389/fcvm.2021.775471)

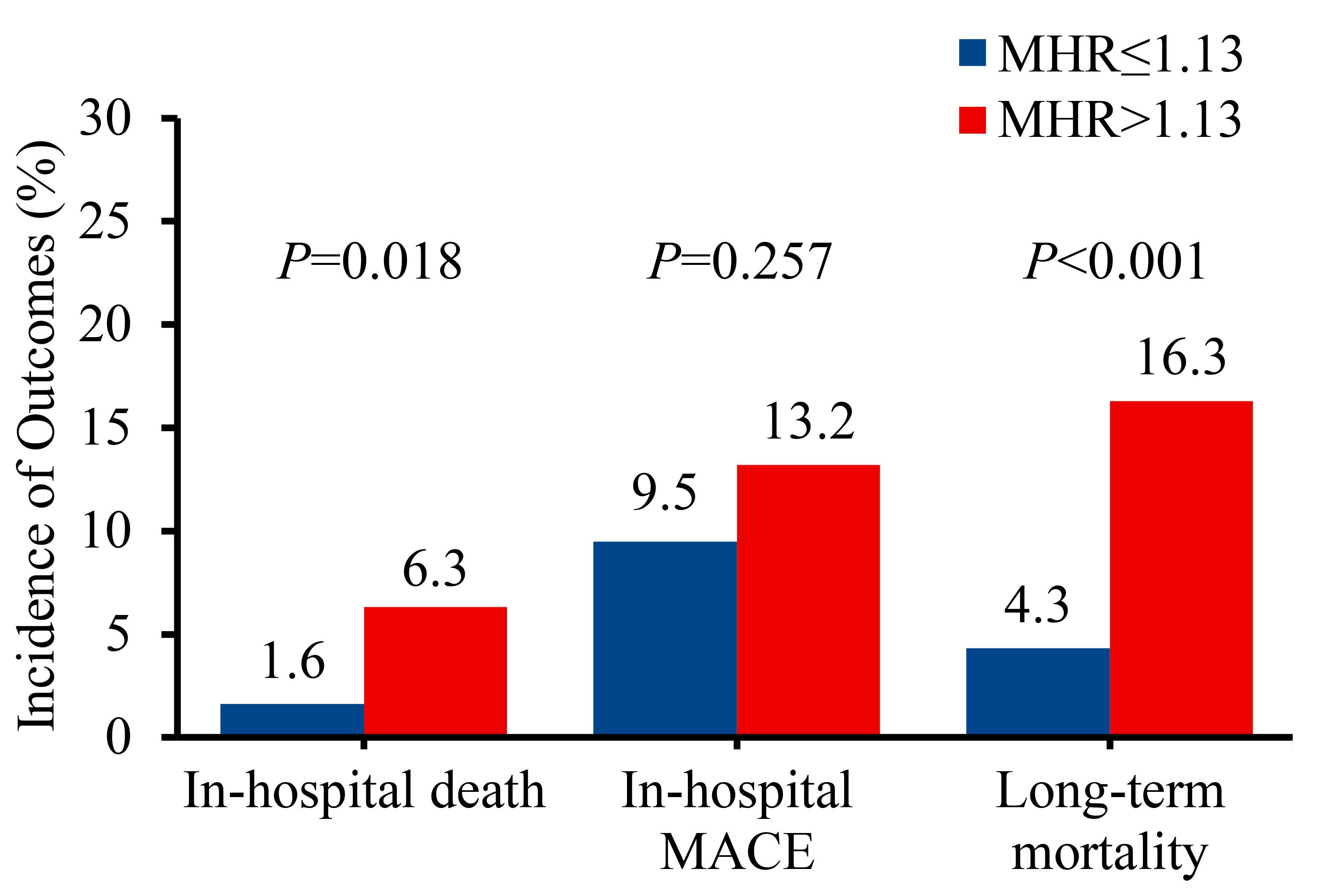

Supplement: Supplementary Figure 1 — Prevalence of adverse events after propensity score matching. MACE, major adverse clinical events; MHR, monocyte to high-density lipoprotein ratio. [file Image_1.TIF]

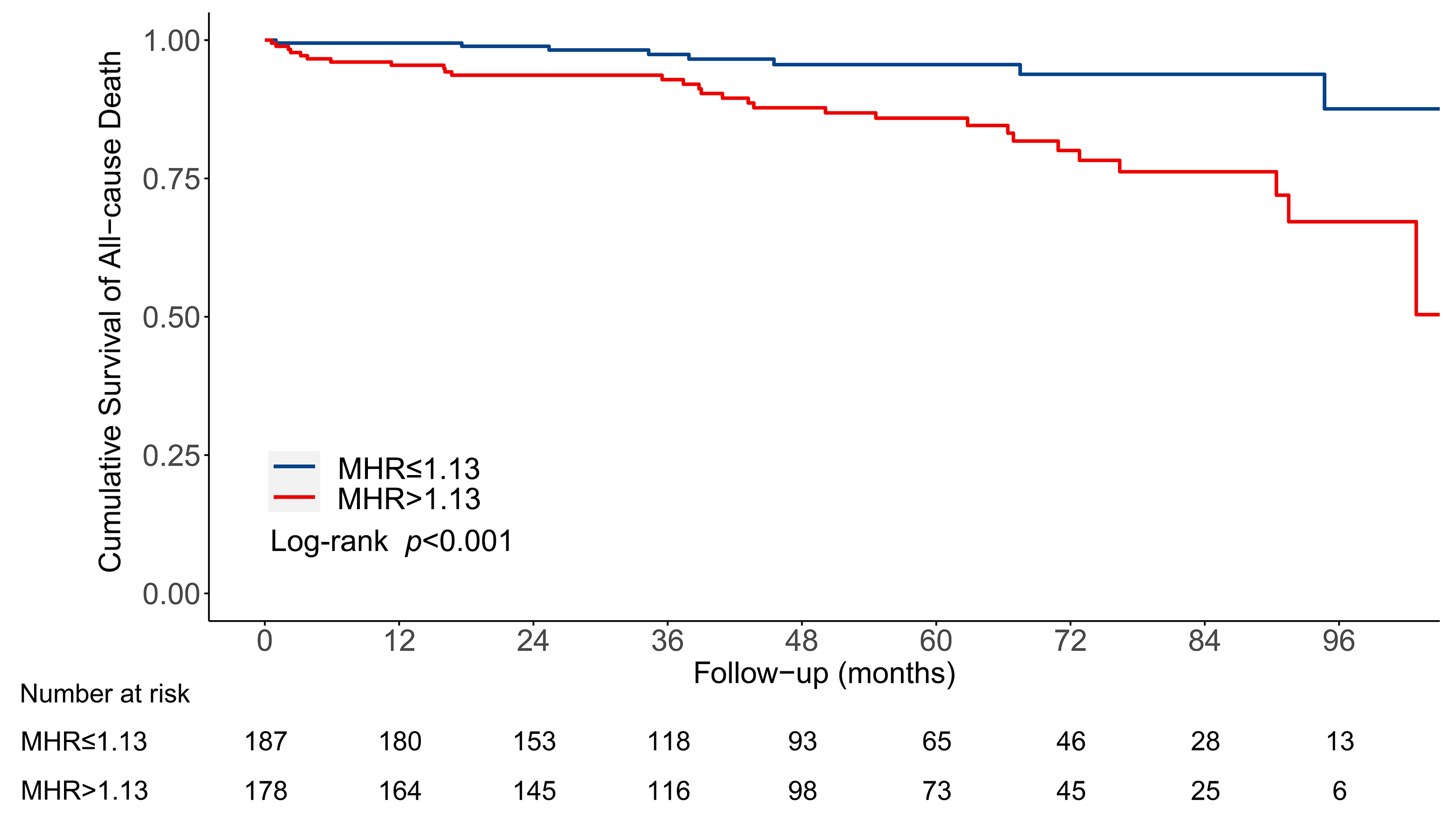

Supplement: Supplementary Figure 2 — Kaplan-Meier curve for cumulative survival rates of long-term mortality after propensity score matching. MHR, monocyte to high-density lipoprotein ratio. [file Image_2.TIF]
